# Memory-Efficient Mapping Based on Geometry-Aware Adaptive Gaussian Control for LiDAR Localization

**DOI:** 10.3390/s26196249

**Published:** 2026-10-01

**Authors:** Hyeonseung Lee, Seongjun Kim, Chansoo Kim

**Affiliations:** 1 Department of Artificial Intelligence Convergence, Chonnam National University, Gwangju 61186, Republic of Korea; leehys4297@jnu.ac.kr (H.L.); wns913@jnu.ac.kr (S.K.); 2 Department of Future Mobility, Chonnam National University, Gwangju 61186, Republic of Korea

**Keywords:** Gaussian map representation, adaptive Gaussian control, geometry-aware optimization, compact map representation, geometry-aware Gaussian mapping, localization, autonomous driving

## Abstract

Efficient map representations are crucial for LiDAR-based localization, as they directly influence localization accuracy and memory efficiency. Among existing map representations, probabilistic and Gaussian-based maps have attracted considerable attention owing to their compact representation and efficient localization capability. However, existing approaches have limited ability to adaptively capture local geometric complexity while maintaining compact map representations. To construct a compact and localizable map representation for LiDAR-based localization, this paper proposes a geometry-aware adaptive Gaussian mapping framework based on geometry-aware optimization and adaptive Gaussian control. The proposed framework constructs a compact LiDAR Gaussian Map by optimizing and adaptively controlling Gaussian primitives according to local geometric characteristics. Experimental results demonstrate superior map compression compared with existing Gaussian-based map representations while achieving localization performance comparable to or better than that of the conventional Normal Distributions Transform localization pipeline. The proposed framework reduces map storage requirements by up to 75.7% while achieving localization accuracy comparable to or better than conventional NDT, demonstrating that localization-relevant geometric constraints can be preserved with fewer Gaussian primitives.

## 1. Introduction

With the rapid advancement and commercialization of autonomous driving technologies, the demand for accurate and memory-efficient spatial representations of large-scale indoor and outdoor environments has continued to increase [1,2]. Such spatial information serves as a fundamental basis for precise localization, enabling autonomous systems to reliably estimate their positions during navigation [2,3]. In map-based localization systems, pre-built high-definition maps provide the reference framework for pose estimation, making the quality and representational capability of the map critical factors affecting localization performance [1,3]. However, storing complete large-scale prior maps onboard can impose substantial storage requirements, while transmitting or streaming regional map data on demand requires compact map files to reduce network bandwidth and data-transfer overhead. Therefore, developing compact map representations that preserve localization-relevant structural information while enabling accurate and computationally efficient localization is a key challenge for practical autonomous driving systems [2,4].

**Figure 1:**
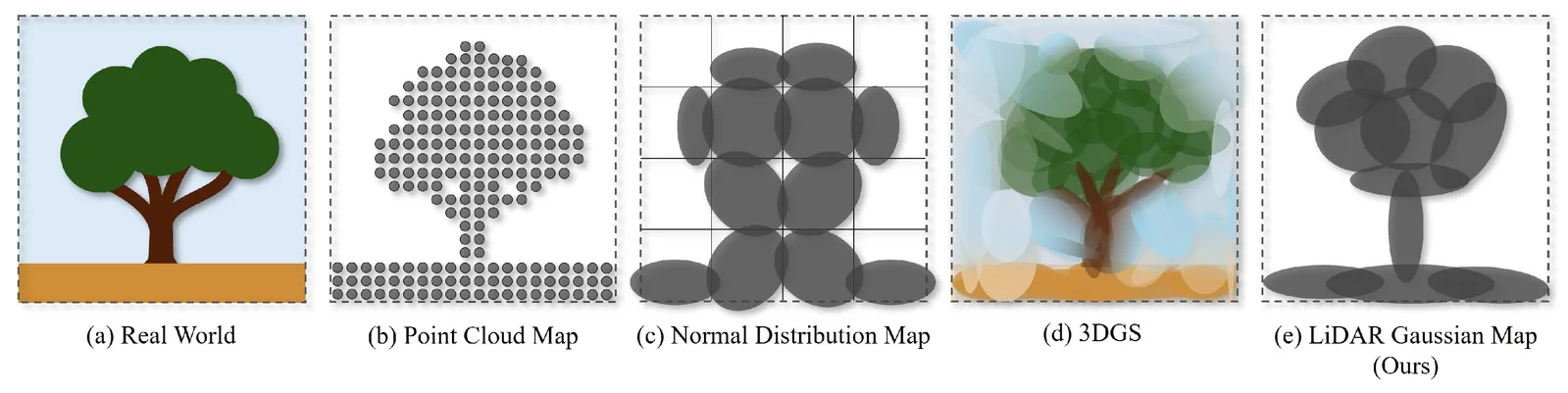
Conceptual comparison of representative map representations, including point-cloud maps, normal distribution maps, 3D Gaussian splatting, and the proposed LiDAR Gaussian map.

Point-cloud maps, as illustrated in Figure 1b, are widely adopted in autonomous driving systems owing to their ability to preserve detailed geometric information of the environment [5,6]. However, as the mapped environment expands, dense point-cloud maps accumulate an increasingly large number of points, leading to substantial storage requirements and a larger memory footprint when the map is loaded for localization. To address this issue, probabilistic map representations such as the normal distributions transform (NDT) have been proposed [7,8]. By partitioning the space into voxels and approximating the point distribution within each voxel using a single Gaussian, as illustrated in Figure 1c, NDT can significantly reduce map storage requirements while enabling efficient scan matching for localization.

Despite these advantages, the fixed voxel discretization employed by NDT can limit the preservation of localization-relevant local structures, particularly in regions containing sharp edges, object boundaries, and complex local geometries [9,10]. Since a single Gaussian is used to represent all points within a voxel, the representation capability is constrained when multiple geometric structures coexist within the same spatial region. Therefore, map representations that can adaptively adjust Gaussian primitives according to the geometric complexity of the environment are required rather than relying on a fixed discretization structure.

Recently, 3D Gaussian splatting (3DGS) has attracted significant attention in the field of neural rendering as an effective scene representation technique that models complex environments using a set of continuous Gaussian primitives [11]. In 3DGS, each primitive is represented by a continuous center, anisotropic scale, and orientation, which define an ellipsoidal support region in 3D space, as illustrated in Figure 1d. Unlike voxel-wise Gaussian representations such as NDT, these primitives are not tied to fixed grid cells, allowing their positions and spatial extents to be adjusted according to local scene structures. In addition, its adaptive density control mechanism adjusts the number and distribution of Gaussian primitives according to scene complexity [11]. These properties are attractive for LiDAR map representation because Gaussian primitives can summarize local point distributions, and their adaptive allocation provides a basis for retaining local geometric structures without uniformly increasing map density.

However, existing 3DGS methods are fundamentally designed for visual rendering and image reconstruction tasks [12,13]. Consequently, the generation and optimization of Gaussian primitives are primarily driven by rendering objectives rather than geometric consistency. As a result, Gaussian primitives may be allocated to regions that do not correspond to actual geometric structures, and the adaptive density control process is guided by rendering errors rather than structural characteristics of point clouds. These properties limit the direct applicability of 3DGS to LiDAR-based localization, where stable localization-relevant geometric constraints are of primary importance.

To address these limitations, this paper proposes a geometry-aware adaptive Gaussian mapping framework that reinterprets the adaptive density control concept of 3DGS for LiDAR-based geometric mapping. The proposed method represents an input point cloud as a set of 3D Gaussian primitives and optimizes their positions, scales, and orientations through a point-to-Gaussian geometry-aware optimization process. In addition, a geometry-aware adaptive Gaussian control strategy dynamically adjusts the density and distribution of Gaussian primitives according to the geometric complexity of the environment, eliminating the dependence on a fixed discretization structure. Consequently, the proposed framework generates a compact Gaussian map that retains sufficient geometric constraints for LiDAR localization while maintaining memory efficiency.

This study presents a geometry-aware adaptive Gaussian mapping framework for constructing compact LiDAR Gaussian maps while preserving localization-relevant geometric constraints in large-scale autonomous driving environments. The main contributions of this paper are as follows.

- **Geometry-Aware Adaptive Gaussian Control:** We propose a geometry-aware adaptive Gaussian control strategy for LiDAR Gaussian mapping. The proposed framework optimizes Gaussian primitives through point-to-Gaussian geometric constraints while adaptively regulating their density and spatial distribution using split, clone, merge, prune, and create-in-learning operations according to local geometric complexity.
- **Compact and Localizable Gaussian Map Representation:** The proposed framework generates compact and localizable Gaussian maps by retaining scan-matching-relevant geometric information while reducing redundant primitives. Experimental results demonstrate that the generated maps support LiDAR scan-matching localization with performance comparable to or better than that of conventional NDT maps while reducing map storage requirements by up to 75.7%.

The remainder of this paper is organized as follows. Section 2 reviews related work on probabilistic map representations and Gaussian-based scene representations. Section 3 presents the proposed geometry-aware adaptive Gaussian mapping framework. Section 4 evaluates the proposed method through map compression, localization, map-resolution trade-off analysis, and ablation studies. Finally, Section 5 concludes the paper and discusses future work.

## 2. Related Work

### 2.1. LiDAR-Based Map Representation for Localization

LiDAR-based localization estimates vehicle pose by registering the current point cloud acquired from onboard sensors to a pre-built map. Therefore, the representational capability and structural characteristics of the map play a critical role in determining both registration accuracy and computational efficiency. Accordingly, various map-representation methods have been proposed to effectively preserve environmental geometry while maintaining efficient storage and processing.

Early studies directly employed dense point-cloud maps to preserve the geometric structure of real-world environments as accurately as possible. Point cloud-based maps have since been widely adopted in autonomous driving applications [14,15,16,17] owing to their ability to retain detailed geometric information of the environment. However, dense point-cloud maps incur rapidly increasing storage requirements as the environment scale grows [18]. In addition, localization using dense point-cloud maps typically requires extensive point searches and correspondence matching, resulting in substantial computational costs. For these reasons, probabilistic map representation approaches have attracted increasing attention, as they can provide higher memory efficiency while preserving the geometric characteristics of point cloud data.

### 2.2. Probabilistic Map Representation

To reduce the storage cost and computational complexity of point-cloud maps, probabilistic map representations have been widely studied. Instead of storing individual points, these methods model point distributions using probability density functions. This enables more compact map representations while preserving geometric information required for localization.

A representative example is NDT. Biber and Strasser [7] first proposed NDT for point cloud registration by representing spatial information using Gaussian distributions. Magnusson [19] later extended this concept to three-dimensional environments. In 3D NDT, the space is partitioned into fixed voxels, and the point distribution within each voxel is approximated by a single Gaussian distribution. Owing to its efficient distribution-based scan matching framework, NDT has been widely adopted for autonomous driving localization [20,21].

Recent studies have further improved probabilistic voxel-based representations by enhancing local geometric modeling while preserving the efficiency of voxel-based maps. For example, VoxelMap [22] represents local planar structures using probabilistic adaptive voxel primitives, enabling more accurate geometric modeling and online LiDAR odometry than conventional NDT while maintaining the probabilistic mapping framework.

Beyond adaptive voxel representations, several other extensions have also been proposed. Representative examples include the Gaussian mixture model (GMM)-based map representations [23,24], multi-resolution voxel maps [25], and occupancy-based probabilistic maps [26]. These approaches aim to represent environmental structures more accurately through multi-modal distributions or hierarchical spatial representations.

However, most existing methods rely on structured spatial partitioning schemes such as grids or tree-based data structures. Gaussian distributions are assigned within predefined spatial regions regardless of the local geometric complexity of the environment. As a result, object boundaries, sharp edges, and complex structural variations are often difficult to represent with a compact fixed-resolution structure. Increasing the spatial resolution can improve local detail retention, but also increases memory consumption. This creates a trade-off between localization-relevant detail retention and memory efficiency.

Consequently, although probabilistic map representations provide high memory efficiency, their ability to represent complex geometric structures remains limited. Recent advances in neural rendering have introduced scene representation methods based on continuous Gaussian primitives. In particular, 3DGS [11] has demonstrated the ability to model complex scenes with high representational fidelity. Gaussian-based representations may provide a promising direction for alleviating some limitations of conventional probabilistic map representations.

### 2.3. Adaptive Gaussian Representation and 3D Gaussian Splatting

3DGS [11] is a representative explicit scene representation method that models a scene using a set of 3D Gaussian primitives. Each Gaussian contains position, covariance, color, and opacity parameters. By employing a Gaussian splatting-based rendering, 3DGS achieves both high rendering quality and real-time performance. Consequently, it has recently gained significant attention in the field of neural rendering.

A key feature of 3DGS is its adaptive density control mechanism. Through split, clone, and pruning operations, the number and density of Gaussian primitives can be adjusted according to the structural complexity of the scene. This enables Gaussian primitives to be distributed adaptively without relying on a fixed spatial partitioning structure. Consequently, 3DGS differs fundamentally from conventional grid-based representations.

More recently, 3DGS-based representations have been explored for a variety of robotics and autonomous driving tasks, including LiDAR simultaneous localization and mapping (SLAM) [27], visual–inertial SLAM [28], reconstruction [29], and localization [30]. In particular, Splat-LOAM [31] demonstrated that Gaussian splatting can be successfully integrated into LiDAR-based odometry and mapping, extending Gaussian representations beyond image-based scene reconstruction. These studies have demonstrated the potential of Gaussian primitive-based representations for spatial modeling. In particular, Gaussian primitives provide a continuous and flexible representation of three-dimensional environments, making them promising for mapping and scene representation tasks.

However, most existing 3DGS-based methods are primarily designed for rendering, reconstruction, or SLAM applications rather than optimizing Gaussian primitives specifically for LiDAR-based localization. Consequently, the geometric characteristics required for robust point-to-Gaussian scan matching are not explicitly considered during Gaussian generation and optimization [11,31,32].

Therefore, to utilize the adaptive density control mechanism of 3DGS for LiDAR-based geometric mapping and localization, the rendering-oriented optimization framework must be reinterpreted as a geometry-aware optimization framework that directly exploits the geometric characteristics of LiDAR point clouds.

## 3. Method

### 3.1. Overall Framework

This paper proposes a geometry-aware adaptive Gaussian mapping framework for constructing compact Gaussian maps from LiDAR point clouds. The proposed framework converts input point clouds into sets of Gaussian primitives and iteratively refines their attributes through geometry-aware optimization and adaptive Gaussian control. The resulting Gaussian map preserves geometric information required for scan matching-based localization while reducing map complexity.

**Figure 2:**
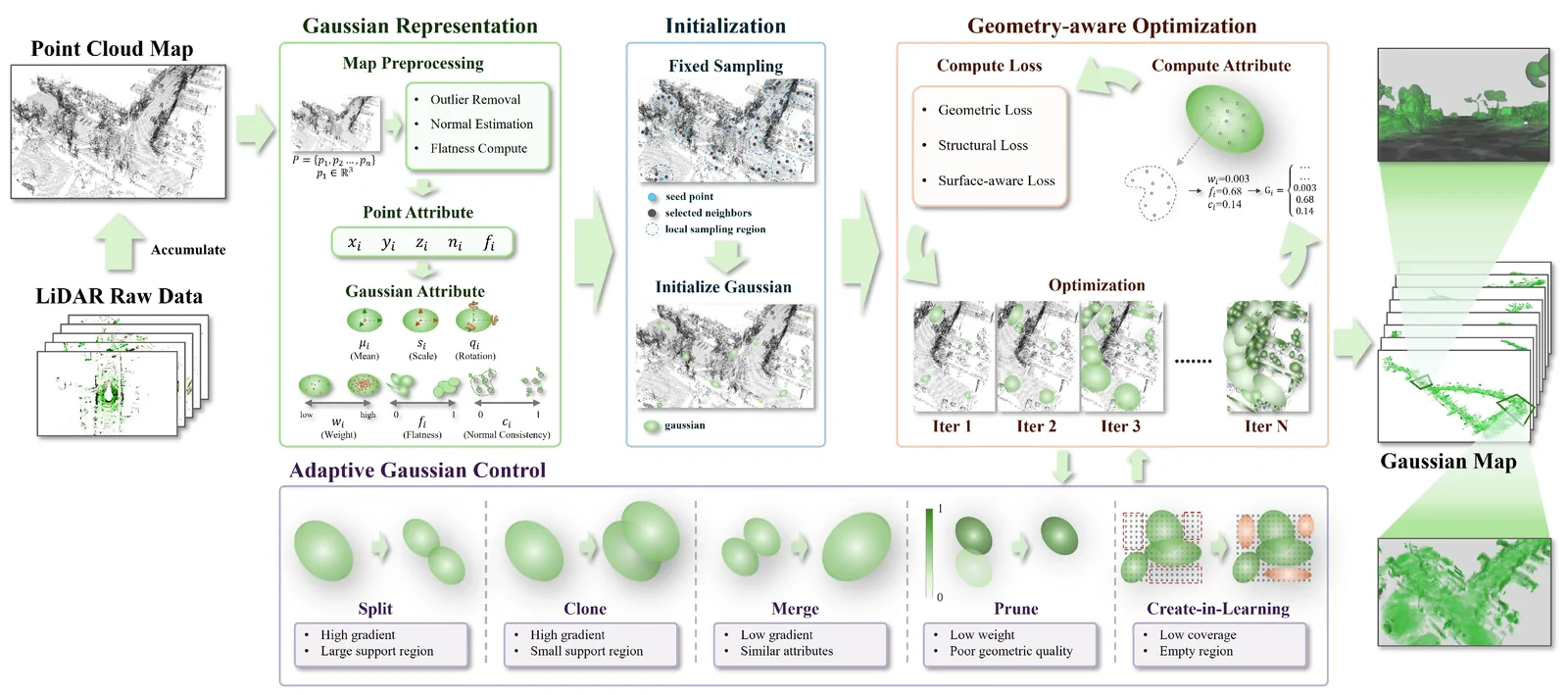
Overall framework of the proposed geometry-aware adaptive Gaussian mapping. The input point cloud is first augmented with geometric attributes, followed by Gaussian initialization and geometry-aware optimization. During optimization, Gaussian attributes are iteratively updated and adaptive control operations, including split, clone, merge, prune, and create-in-learning, are performed to balance representation capability and map compactness.

As illustrated in Figure 2, the overall pipeline consists of Gaussian representation, Gaussian initialization, geometry-aware optimization, and adaptive Gaussian control. First, local geometric attributes, including normal vectors and flatness values, are estimated from the input point cloud. These attributes are used to initialize Gaussian primitives with position, scale, orientation, and geometric properties.

The initialized Gaussian set is then optimized according to its geometric relationship with the input point cloud. During optimization, adaptive Gaussian control dynamically refines the Gaussian representation according to the local geometric complexity.

The input point cloud is defined as

(1) $$P=p_{1},p_{2},\ldots ,p_{N},\;p_{j}\in R^{3}$$

where *N* denotes the number of input points. The resulting Gaussian map is represented as

(2) $$G=G_{1},G_{2},\ldots ,G_{M}$$

where *M* denotes the number of Gaussian primitives. Each Gaussian primitive contains position, scale, orientation, and local geometric attributes. A detailed definition of the Gaussian representation is provided in Section 3.2.

### 3.2. Gaussian Representation

To construct the Gaussian representation, the input point cloud is first preprocessed to estimate local geometric attributes. Following [31], outlier removal, normal vector estimation, and point-wise flatness computation are performed.

Each Gaussian primitive $G_{i}$ represents a local geometric structure and is characterized by its mean $\mu_{i}$, covariance $\Sigma_{i}$, weight $w_{i}$, flatness $f_{i}$, and normal consistency $c_{i}$. The mean and covariance describe the spatial distribution of the Gaussian, while the additional attributes are introduced to capture local geometric characteristics and support the subsequent geometry-aware optimization and adaptive Gaussian control processes. The Gaussian weight reflects the relative importance of the represented local structure, whereas flatness and normal consistency characterize local surface properties.

Flatness is computed from the covariance eigenvalues $\lambda_{1}$, $\lambda_{2}$, and $\lambda_{3}$ [32] as

(3) $$f_{i}=\frac{\lambda_{2}-\lambda_{3}}{\lambda_{1}}$$

Normal consistency quantifies the alignment of normal vectors within the support region of a Gaussian. The support region of a Gaussian primitive is defined as its 3σ confidence ellipsoid derived from the covariance matrix.

For each Gaussian primitive $G_{i}$, a normal structure tensor is constructed from the surface normals of the associated points:

(4) $$S_{i}=\frac{1}{K_{i}}\sum_{k=1}^{K_{i}}n_{ik}n_{ik}^{T}$$

Let $\eta_{1}\geq \eta_{2}\geq \eta_{3}$ denote the eigenvalues of the structure tensor $S_{i}$. A normal consistency score is then computed as

(5) $$c_{i}=\frac{\eta_{1}-\eta_{2}}{\eta_{1}+\eta_{2}+\eta_{3}+\epsilon }$$

A high normal consistency value indicates that the local structure exhibits a consistent surface orientation.

Consequently, each Gaussian primitive simultaneously encodes spatial distribution, orientation, structural importance, and local geometric characteristics. These attributes provide the foundation for the subsequent geometry-aware optimization and adaptive Gaussian control processes.

### 3.3. Gaussian Initialization

The objective of the Gaussian initialization stage is to transform the input point cloud into an initial set of Gaussian primitives that provides a stable starting point for the geometry-aware optimization and adaptive Gaussian control.

In the proposed framework, a deterministic fixed-sampling initialization strategy is employed. First, all points are sorted according to their spatial coordinates to ensure deterministic seed selection. An unused point is selected as a seed point, and up to $N_{\max}^{\mathrm{init}}$ neighboring unused points within the initialization radius $r_{\mathrm{init}}$ are collected to form a Gaussian candidate. A Gaussian primitive is generated only when the number of collected points satisfies the minimum support condition $N_{\min}^{\mathrm{init}}$.

After each Gaussian primitive is initialized, the points used for its creation and the points within the exclusion radius $r_{\mathrm{excl}}$ are excluded from the subsequent seed selection. This prevents redundant initialization in the same local region and encourages a spatially distributed initial Gaussian set.

The mean and covariance of each Gaussian primitive are estimated from the selected points, and the resulting Gaussian primitives are subsequently refined through geometry-aware optimization.

### 3.4. Geometry-Aware Optimization

To jointly consider geometric alignment, structural compactness, and surface-aware regularization, the proposed optimization consists of three loss components: geometric alignment loss, structural regularization loss, and surface-aware regularization loss.

The overall optimization objective is defined as follows:

(6) $$L_{\mathrm{total}}=L_{\mathrm{geo}}+L_{\mathrm{struct}}+L_{\mathrm{surf}}$$

#### 3.4.1. Geometric Alignment Loss

The geometric alignment loss is designed to improve the geometric alignment between the input point cloud and the Gaussian primitives. The loss is defined as follows:

(7) $$L_{\mathrm{geo}}=\alpha_{\mathrm{mah}}L_{\mathrm{mah}}+\alpha_{\mathrm{ch}}L_{\mathrm{ch}}$$

where $\alpha_{\mathrm{mah}}$ and $\alpha_{\mathrm{ch}}$ are weighting factors associated with each loss term.

(1) **Mahalanobis Distance**

Mahalanobis distance is widely used in NDT and GMM-based registration methods because it can measure point-to-distribution consistency while considering the covariance structure of local geometry [19]. Motivated by this concept, the proposed framework introduces a Mahalanobis alignment term in the geometric loss to encourage each Gaussian primitive to align with the input point cloud according to its anisotropic shape.

For a point $p_{j}$ and a Gaussian primitive $G_{i}$, the squared Mahalanobis distance is defined as

(8) $$d_{ij}^{\mathrm{mah}}={\left(p_{j}-\mu_{i}\right)}^{T}\Sigma_{i}^{-1}\left(p_{j}-\mu_{i}\right),$$

where $\mu_{i}$ denotes the mean of $G_{i}$. The covariance matrix is reconstructed from the scale and rotation parameters as

(9) $$\Sigma_{i}=R\left(q_{i}\right)\mathrm{diag}\left(s_{i}^{2}\right)R{\left(q_{i}\right)}^{T},$$

where $R(q_{i})$ is the rotation matrix converted from the quaternion $q_{i}$, and $s_{i}$ denotes the anisotropic scale.

Instead of using a hard nearest-Gaussian assignment, the proposed method applies adaptive candidate filtering followed by soft assignment. For each point $p_{j}$, the valid Gaussian candidate set is defined as

(10) $$C_{j}=\left\{i\;|\;{∥p_{j}-\mu_{i}∥}_{2}\leq r_{i}^{\mathrm{eff}}\right\},$$

where

(11) $$r_{i}^{\mathrm{eff}}=\sqrt{\lambda_{\max}\left(\Sigma_{i}\right)}+1.$$

Here, $\lambda_{\max}\left(\Sigma_{i}\right)$ denotes the largest eigenvalue of $\Sigma_{i}$. This adaptive radius allows each Gaussian primitive to define its candidate region according to its spatial extent.

For the candidate Gaussians in $C_{j}$, the soft assignment weight is computed using an inverse-distance softmax:

(12) $$\alpha_{ij}=\frac{\exp\left({\left(d_{ij}^{\mathrm{mah}}+\epsilon \right)}^{-1}\right)}{\sum_{k\in C_{j}}\exp\left({\left(d_{kj}^{\mathrm{mah}}+\epsilon \right)}^{-1}\right)},\;i\in C_{j},$$

The Mahalanobis alignment term used in the geometric loss is then defined as

(13) $$L_{\mathrm{mah}}=\frac{1}{|P_{v}|}\sum_{p_{j}\in P_{v}}\sum_{i\in C_{j}}\alpha_{ij}d_{ij}^{\mathrm{mah}},$$

where $P_{v}$ denotes the set of input points that have at least one valid Gaussian candidate.

The Mahalanobis loss can be viewed as a covariance-aware point-to-Gaussian residual accumulated over the soft candidate associations. Therefore, it serves as the primary alignment term for refining Gaussian primitives while avoiding unstable hard correspondences during Gaussian map optimization. From an optimization perspective, for a fixed candidate set $C_{j}$, the loss provides gradients to the Gaussian mean $\mu_{i}$, scale $s_{i}$, and rotation $q_{i}$ through $d_{ij}^{\mathrm{mah}}$ and the covariance parameterization in Equation (9), whereas the construction of $C_{j}$ is treated as a non-differentiable selection step.

(2) **Chamfer Distance**

To encourage bidirectional geometric coverage between the input point cloud and the Gaussian map, a Chamfer distance term is employed. Chamfer distance is widely used for evaluating geometric similarity between point sets in point-set matching and shape reconstruction tasks [33].

Let

$$M_{G}=\left\{\mu_{1},\mu_{2},\ldots ,\mu_{M}\right\}$$

denote the set of Gaussian means, where *M* is the number of Gaussian primitives. The Chamfer loss between the input point set *P* and the Gaussian mean set $M_{G}$ is defined as

(14) $$L_{\mathrm{ch}}=\frac{1}{N}\sum_{j=1}^{N}\min_{1\leq i\leq M}{∥p_{j}-\mu_{i}∥}_{2}+\frac{1}{M}\sum_{i=1}^{M}\min_{1\leq j\leq N}{∥\mu_{i}-p_{j}∥}_{2}.$$

The first term encourages each input point to be represented by a nearby Gaussian primitive, whereas the second term prevents Gaussian means from being located far from the observed point cloud.

These bidirectional nearest-neighbor associations are used to provide coverage constraints on the Gaussian mean positions. Under fixed nearest-neighbor assignments, the Chamfer loss is piecewise differentiable with respect to the Gaussian means, while the min-based nearest-neighbor selection itself is treated as a non-differentiable matching operation. Thus, the Chamfer loss complements the covariance-aware Mahalanobis loss by preventing Gaussian primitives from drifting away from the input point distribution and by improving bidirectional geometric coverage.

#### 3.4.2. Structural Regularization Loss

During optimization, redundant Gaussian primitives may represent the same local structure, while others may converge to inefficient configurations. To encourage a compact and structurally stable Gaussian representation, a Structural Regularization term is introduced:

(15) $$L_{\mathrm{struct}}=\beta_{\mathrm{ov}}L_{\mathrm{ov}}+\beta_{\mathrm{like}}L_{\mathrm{like}}+\beta_{\mathrm{comp}}L_{\mathrm{comp}}$$

where $\beta_{\mathrm{ov}}$, $\beta_{\mathrm{like}}$, and $\beta_{\mathrm{comp}}$ denote the weighting factors of each regularization term.

(1) **Overlap Regularization**

To suppress excessive overlap between Gaussian primitives, an overlap regularization term is introduced. For each Gaussian pair, bidirectional Mahalanobis distances are computed using Equation (8). If either Gaussian center lies within the support region of the other, an overlap penalty is assigned.

(16) $$o_{ij}=\max\left(0,\tau_{\mathrm{ov}}-\min\left(d_{i\to j}^{\mathrm{mah}},d_{j\to i}^{\mathrm{mah}}\right)\right),$$

where $d_{i\to j}^{\mathrm{mah}}$ denotes the Mahalanobis distance from the center of $G_{i}$ to $G_{j}$, and $d_{j\to i}^{\mathrm{mah}}$ denotes the reverse direction. The threshold $\tau_{\mathrm{ov}}$ controls the allowable overlap between Gaussian primitives.

The pairwise overlap loss is then defined as

(17) $$L_{\mathrm{pair}}=\sum_{i<j}o_{ij}^{2}.$$

To prevent covariance collapse, a variance lower-bound penalty is additionally applied. Let $\lambda_{i,k}$ denote the *k*-th eigenvalue of the covariance matrix $\Sigma_{i}$. The variance penalty is defined as

(18) $$L_{\mathrm{var}}=\frac{1}{M}\sum_{i=1}^{M}\sum_{k=1}^{3}\max{\left(0,\tau_{\mathrm{var}}-\lambda_{i,k}\right)}^{2},$$

where $\tau_{\mathrm{var}}$ is the minimum allowable variance threshold. Since the covariance matrix is parameterized as $\Sigma_{i}=R\left(q_{i}\right)\mathrm{diag}\left(s_{i}^{2}\right)R{\left(q_{i}\right)}^{T}$, the eigenvalues correspond to the squared scale values of each Gaussian primitive.

The final overlap regularization loss is defined as

(19) $$L_{\mathrm{ov}}=L_{\mathrm{pair}}+L_{\mathrm{var}}.$$

For Gaussian pairs included in the overlap computation, the pairwise overlap term is piecewise differentiable with respect to the Gaussian mean, scale, and rotation through the bidirectional Mahalanobis distances. Pair selection and threshold-based activation are treated as non-differentiable conditions, while the variance lower-bound term mainly provides gradients to the Gaussian scale parameters.

Overall, the overlap regularization reduces primitive redundancy and correspondence ambiguity by discouraging multiple Gaussian primitives from representing the same local structure, while the variance penalty prevents covariance collapse during optimization.

(2) **Likelihood-based Regularization**

A likelihood-based loss is introduced to encourage the Gaussian primitive set to represent the overall spatial distribution of the input point cloud. Inspired by the likelihood formulation of GMMs [34], the negative log-likelihood term is defined as

(20) $$L_{\mathrm{nll}}=-\frac{1}{N}\sum_{j=1}^{N}\log\left(\sum_{i=1}^{M}w_{i}N\left(p_{j}|\mu_{i},\Sigma_{i}\right)\right),$$

where $w_{i}$ denotes a non-negative support-related coefficient associated with Gaussian primitive $G_{i}$. In the current implementation, $w_{i}$ is derived from the number of input points contained within the support region of $G_{i}$ and is periodically updated according to the current point-to-Gaussian associations. The coefficient is used as an unnormalized support-related coefficient rather than a softmax-normalized mixture probability.

During backpropagation, the support-related coefficient $w_{i}$ is detached and does not receive gradients. In contrast, the GMM NLL term remains differentiable with respect to the Gaussian mean $\mu_{i}$ and covariance $\Sigma_{i}$, thereby providing gradients to the Gaussian mean, scale, and rotation through the covariance parameterization.

The final likelihood-related loss is defined as

(21) $$L_{\mathrm{like}}=\beta_{\mathrm{nll}}L_{\mathrm{nll}}$$

where $\beta_{\mathrm{nll}}$ denotes the weighting factor of the support-weighted log-density regularization term. Overall, this likelihood-based regularization stabilizes the Gaussian set by evaluating whether the primitives remain consistent with the current point support and overall spatial distribution of the input point cloud.

(3) **Compactness Regularization**

Some Gaussian primitives may occupy excessively large spatial regions while being supported by only a small number of points, resulting in inefficient geometric representations. To address this issue, a compactness regularization term is introduced to balance the spatial extent of a Gaussian primitive and its point support.

For each Gaussian primitive, support points are identified using a predefined Mahalanobis distance threshold. The spatial extent is estimated using the volume of its support region, which is approximated as the 3σ confidence interval ellipsoid derived from the covariance matrix:

(22) $$V_{i}=36\pi s_{i1}s_{i2}s_{i3}$$

Based on the support volume $V_{i}$ and the number of support points $n_{i}$, the compactness score of each Gaussian primitive is defined as follows:

(23) $$C_{i}=\frac{V_{i}}{n_{i}+\epsilon }$$

The final compactness regularization is formulated as a weighted average based on the structural importance of each Gaussian primitive:

(24) $$L_{\mathrm{comp}}=\frac{\sum_{i}w_{i}C_{i}}{\sum_{i}w_{i}+\epsilon }$$

For fixed support assignments, the compactness loss is piecewise differentiable mainly with respect to the Gaussian scale parameters through the support volume $V_{i}$, while the support-point selection, support count $n_{i}$, and support-related coefficient $w_{i}$ are treated as fixed statistics during each optimization step.

Consequently, this term suppresses Gaussian primitives that occupy excessively broad regions with limited point support, improving map compactness while maintaining localization-relevant geometric constraints.

#### 3.4.3. Surface-Aware Regularization Loss

To preserve local surface characteristics, a surface-aware regularization term is introduced. The surface-aware regularization loss is formulated using the projected surface area term as follows:

(25) $$L_{\mathrm{surf}}=\gamma_{\mathrm{area}}L_{\mathrm{area}}$$

where $\gamma_{\mathrm{area}}$ denotes the weighting factor of the projected surface area loss.

(1) **Projected Surface Area Loss**

The projected surface area loss is introduced to regulate the spatial spread of Gaussian primitives with respect to local surface structures. Since real-world environments contain numerous planar regions, Gaussian primitives should form anisotropic distributions that are consistent with the underlying surface geometry.

To quantify the spatial extent of a Gaussian primitive, its covariance matrix is projected onto the three coordinate planes (XY,XZ,YZ), and the projected area is computed as

(26) $$A_{i}=n_{\sigma }^{2}\left(\sqrt{\det(\Sigma_{i}^{XY})}+\sqrt{\det(\Sigma_{i}^{XZ})}+\sqrt{\det(\Sigma_{i}^{YZ})}\right)$$

where $\Sigma_{i}^{XY}$, $\Sigma_{i}^{XZ}$, and $\Sigma_{i}^{YZ}$ denote the projected covariance matrices, and $n_{\sigma }$ controls the support range of the Gaussian primitive.

The projected area is weighted by the Gaussian flatness, yielding the following:

(27) $$L_{\mathrm{area}}=\frac{1}{M}\sum_{i=1}^{M}f_{i}A_{i}$$

where $f_{i}$ denotes the Gaussian flatness defined in Section 3.2. Consequently, stronger regularization is applied to highly planar Gaussian primitives.

Since the projected covariance matrices are derived from $\Sigma_{i}$, this loss is differentiable with respect to the Gaussian scale $s_{i}$ and rotation $q_{i}$, while the flatness coefficient $f_{i}$ is treated as a detached local geometric statistic.

Thus, the projected surface area loss encourages planar Gaussian primitives to maintain surface-aligned anisotropic shapes by penalizing unnecessary spatial expansion that does not contribute to local surface constraints.

### 3.5. Adaptive Gaussian Control

Through geometry-aware optimization, the positions, scales, and orientations of Gaussian primitives are refined to provide stable point-to-Gaussian geometric constraints for LiDAR localization. However, when the number of Gaussian primitives remains fixed, parameter optimization alone cannot fully adapt the representation to varying geometric complexity. To address this limitation, the proposed framework incorporates a geometry-aware adaptive Gaussian control (AGC) strategy inspired by the adaptive density control of [11], which dynamically adjusts the number and distribution of Gaussian primitives according to local geometric characteristics.

AGC performs split, clone, merge, prune, and create-in-learning operations based on the optimization status and geometric properties of Gaussian primitives. Split and clone improve the representation of geometrically complex regions, whereas merge and prune remove redundant Gaussian primitives to improve map compactness. Create-in-learning additionally introduces new Gaussian primitives in underrepresented regions to improve spatial coverage.

For each Gaussian primitive $G_{i}$, the mean gradient magnitude is defined as

(28) $$g_{i}={∥\frac{\partial L_{\mathrm{total}}}{\partial \mu_{i}}∥}_{2}$$

In addition, the Gaussian attributes defined in Section 3.2, including flatness $f_{i}$ and normal consistency $c_{i}$, are used to evaluate local geometric coherence in AGC. The support volume and compactness score defined in Section 3.4.2 are denoted by $V_{i}$ and $C_{i}$, respectively.

#### 3.5.1. Split

**Figure 3:**
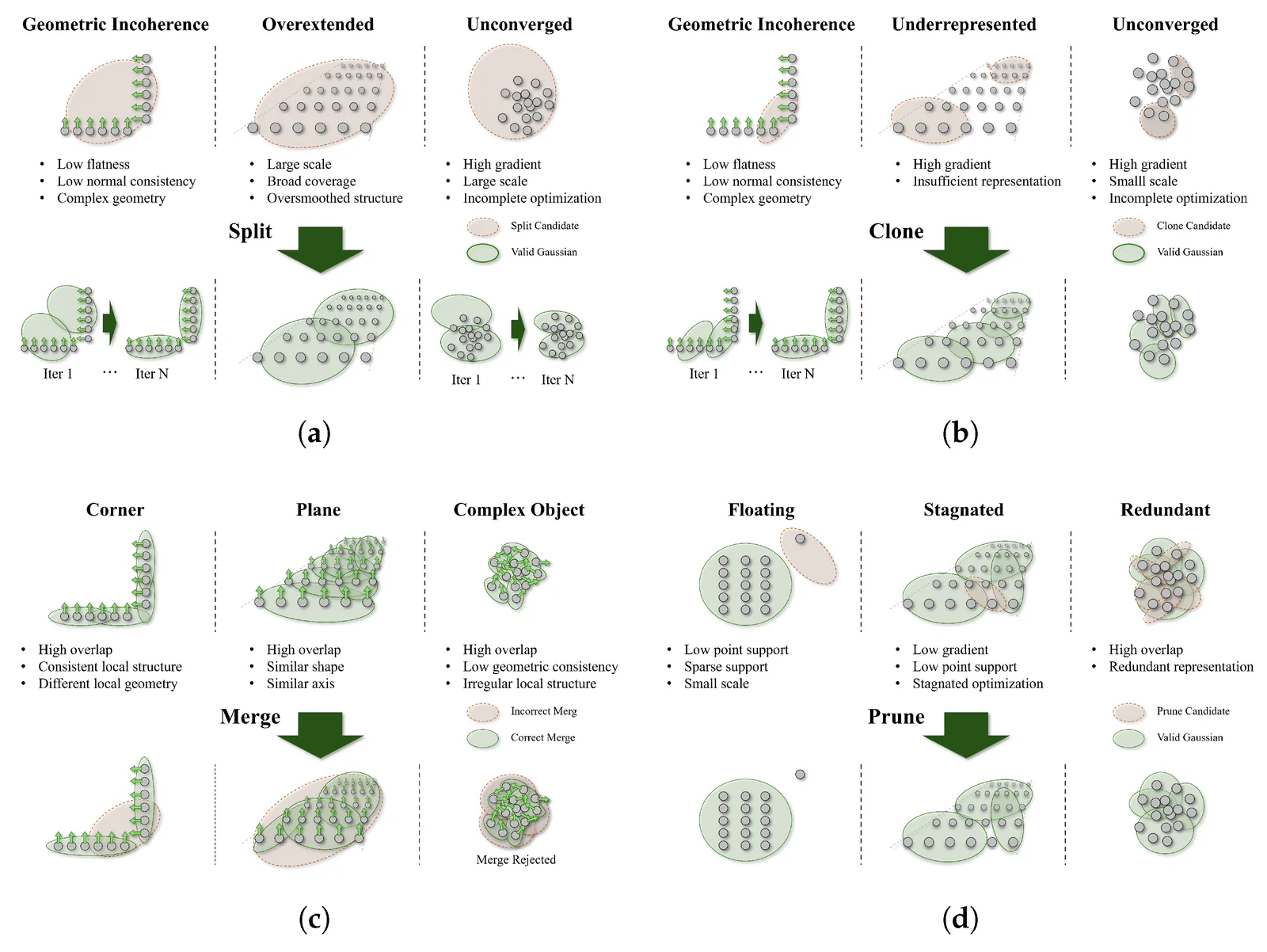
Adaptive Gaussian control operations: (**a**) Split, (**b**) Clone, (**c**) Merge, and (**d**) Prune. Each operation dynamically refines the Gaussian representation according to local geometric characteristics and optimization states.

In the proposed framework, split candidates are selected by jointly considering the optimization status, local geometric coherence, and spatial extent of Gaussian primitives. Figure 3a illustrates representative examples of the split operation. A split is performed when a Gaussian primitive has not sufficiently converged and either exhibits low geometric coherence or occupies an excessively large spatial extent. By replacing a single Gaussian primitive with multiple smaller Gaussian primitives, the local primitive allocation can be refined in geometrically complex regions.

A Gaussian primitive $G_{i}$ is selected for split when the following condition is satisfied:

(29) $$\mathrm{Split}\left(G_{i}\right)=\left\{\begin{array}{cc} 1, & g_{i}\geq \tau_{g}\wedge \left(\Psi_{i}\vee \max\left(s_{i}\right)>\tau_{s}\right)\\ 0, & \mathrm{otherwise} \end{array}\right.$$

where $\Psi_{i}$ denotes a geometry-consistency condition defined from the Gaussian flatness $f_{i}$ and normal consistency $c_{i}$. It is formulated as

(30) $$\Psi_{i}=\left(f_{i}\leq \tau_{f1}\wedge c_{i}\leq \tau_{c1}\wedge \left(f_{i}\leq \tau_{f2}\vee c_{i}\leq \tau_{c2}\right)\right)\vee \left(f_{i}\leq \tau_{f3}\wedge c_{i}\leq \tau_{c3}\right).$$

The condition $\Psi_{i}$ identifies Gaussian primitives with insufficient geometric coherence, where low flatness or low normal consistency indicates that a single Gaussian primitive may not adequately represent the underlying local structure.

When $\max\left(s_{i}\right)>\tau_{s}$, the Gaussian primitive is considered to occupy an excessively large spatial region. Such Gaussian primitives may oversmooth complex local structures and are therefore divided into multiple Gaussian primitives to enable finer primitive allocation.

In addition to these structural conditions, the gradient criterion $g_{i}\geq \tau_{g}$ is required for all split candidates. A large optimization gradient indicates that the Gaussian primitive has not yet sufficiently converged and that additional structural refinement may improve its representation. In the optimization process, the split operation is scheduled from iteration 30 and is applied every 40 iterations.

As a result, Gaussian primitives that remain under-optimized and are either geometrically inconsistent or excessively large are replaced by two smaller child primitives following the adaptive density control strategy of 3DGS [11], enabling finer primitive allocation in locally complex regions. The inserted child primitives are subsequently refined through the optimization loss.

#### 3.5.2. Clone

The clone operation is introduced to increase the local representational capacity while preserving the original Gaussian primitive. Figure 3b illustrates representative examples of the clone operation. Unlike the split operation, clone preserves the original Gaussian primitive and introduces an additional Gaussian primitive to increase local representational capacity in geometrically complex or underrepresented regions. In the proposed framework, clone candidates are selected by jointly considering the optimization status and geometric coherence of Gaussian primitives.

As the candidate conditions of split and clone can partially overlap, the two operations are controlled by separate schedules with different iteration offsets. Clone is applied every 40 iterations with a 20-iteration offset relative to the split schedule. This prevents a Gaussian primitive from being simultaneously processed by both split and clone at the same optimization iteration.

A Gaussian primitive $G_{i}$ is selected for the clone operation when the following condition is satisfied:

(31) $$\mathrm{Clone}\left(G_{i}\right)=\left\{\begin{array}{cc} 1, & g_{i}\geq \tau_{g}\wedge \Psi_{i}\\ 0, & \mathrm{otherwise} \end{array}\right.$$

where $\Psi_{i}$ denotes the geometry-consistency condition defined from the Gaussian flatness $f_{i}$ and normal consistency $c_{i}$.

The condition $\Psi_{i}$ identifies Gaussian primitives that exhibit low geometric coherence, where low flatness or low normal consistency indicates that a single Gaussian primitive may not adequately represent the underlying local structure. Such Gaussian primitives are likely to correspond to underrepresented local regions, where the current primitive allocation may be insufficient for representing localization-relevant local constraints.

In addition to the geometric coherence condition, the gradient criterion $g_{i}\geq \tau_{g}$ is required for all clone candidates. A large optimization gradient indicates that the Gaussian primitive has not yet sufficiently converged and may therefore benefit from additional representational capacity.

When a Gaussian primitive is selected for clone, the original primitive is retained and one additional Gaussian primitive is inserted into the Gaussian set following the adaptive density control strategy of 3DGS [11]. The newly inserted primitive is then refined in subsequent optimization iterations, thereby increasing the local representational capacity without removing the source Gaussian.

#### 3.5.3. Merge

The merge operation is performed when neighboring Gaussian primitives redundantly represent the same local geometric structure. Figure 3c illustrates the merge candidate evaluation process. The proposed framework does not merge Gaussian primitives solely based on spatial proximity or overlap. Instead, overlap and geometric consistency are jointly considered when determining whether two Gaussian primitives should be merged.

To evaluate the degree of redundancy between Gaussian primitives, an overlap score is first computed using the sets of points contained within their support regions. For two Gaussian primitives $G_{i}$ and $G_{j}$, the overlap score is defined as

(32) $$P_{G_{i}}=\left\{p\in P\;|\;\sum_{a=1}^{3}{\left(\frac{{\left[R_{i}^{T}\left(p-\mu_{i}\right)\right]}_{a}}{\sigma_{m}s_{i,a}}\right)}^{2}\leq 1\right\}$$

(33) $$O_{ij}^{\mathrm{merge}}=\frac{\left|P_{G_{i}}\cap P_{G_{j}}\right|}{\min\left(\left|P_{G_{i}}\right|,\left|P_{G_{j}}\right|\right)}$$

where P denotes the input point set, and $P_{G_{i}}$ and $P_{G_{j}}$ denote the subsets of P contained within the support regions of Gaussian primitives $G_{i}$ and $G_{j}$, respectively. A higher overlap score indicates that the two Gaussian primitives are likely to redundantly represent the same local geometric structure.

To prevent geometrically dissimilar Gaussian primitives from being merged, merge candidates are first searched among the $K_{\mathrm{merge}}$ nearest neighboring Gaussian primitives. When either Gaussian primitive exhibits a sufficiently planar structure, the directions of their smallest-variance axes are required to be sufficiently consistent. Let $\Pi_{i}$ denote the planarity indicator of $G_{i}$, defined as

(34) $$\Pi_{i}=I\left(6s_{i,\min}\leq \tau_{\mathrm{plane}}\right),$$

where $6s_{i,\min}$ represents the full 3σ extent along the smallest-variance axis of $G_{i}$, and $\tau_{\mathrm{plane}}$ is the thickness threshold used to identify sufficiently planar Gaussian primitives. The indicator $\Pi_{j}$ is defined analogously for $G_{j}$. The geometric consistency criterion $\Phi_{ij}$ is defined as

(35) $$\Phi_{ij}=\left\{\begin{array}{cc} \left|u_{i}^{T}u_{j}\right|\geq \tau_{\mathrm{dir}},\; & \mathrm{if}\;\Pi_{i}\vee \Pi_{j},\\ 1,\; & \mathrm{otherwise}, \end{array}\right.$$

where $u_{i}$ and $u_{j}$ denote the unit vectors corresponding to the smallest-variance axes of $G_{i}$ and $G_{j}$, respectively, and $\tau_{\mathrm{dir}}$ is the directional similarity threshold.

A Gaussian pair is selected as a merge candidate when the overlap score exceeds a predefined threshold and the two Gaussian primitives exhibit sufficient geometric consistency:

(36) $$\mathrm{Merge}\left(G_{i},G_{j}\right)=\left\{\begin{array}{cc} 1, & \left(O_{ij}^{\mathrm{merge}}>\tau_{m}\right)\wedge \Phi_{ij}\\ 0, & \mathrm{otherwise} \end{array}\right.$$

The merge operation is scheduled after iteration 70 and is periodically applied every 60 iterations with a 32-iteration offset. This delayed application prevents premature merging before the local Gaussian structures are sufficiently stabilized.

When one or more Gaussian pairs satisfy the merge condition, connected merge groups are formed from the selected pairs. For each merge group $I_{\mathrm{merge}}$, all Gaussian primitives in the group are removed and replaced by a single representative Gaussian. The mean of the merged Gaussian is computed as the support-weighted average of the selected Gaussian means:

(37) $$\mu_{\mathrm{merge}}=\frac{1}{W_{\mathrm{merge}}}\sum_{i\in I_{\mathrm{merge}}}w_{i}\mu_{i},\;W_{\mathrm{merge}}=\sum_{i\in I_{\mathrm{merge}}}w_{i}.$$

The covariance of the merged Gaussian is computed by combining the within-Gaussian covariance and the between-mean covariance of the merge group:

(38) $$\Sigma_{\mathrm{merge}}=\frac{1}{W_{\mathrm{merge}}}\sum_{i\in I_{\mathrm{merge}}}w_{i}\Sigma_{i}+\frac{1}{W_{\mathrm{merge}}}\sum_{i\in I_{\mathrm{merge}}}w_{i}\left(\mu_{i}-\mu_{\mathrm{merge}}\right){\left(\mu_{i}-\mu_{\mathrm{merge}}\right)}^{T}.$$

The scale and rotation of the merged Gaussian are then recovered from the eigendecomposition of $\Sigma_{\mathrm{merge}}$, and the merged primitive is refined in subsequent optimization iterations.

By jointly considering overlap and geometric consistency, the proposed merge operation consolidates redundant Gaussian primitives in planar and corner-like structures while preventing incorrect merging in geometrically complex regions. As a result, redundant primitives are replaced by a representative Gaussian that preserves the spatial extent of the merged local structure, thereby maintaining localization-relevant geometric information while improving map compactness.

#### 3.5.4. Prune

The prune operation removes weakly supported or locally redundant Gaussian primitives to improve map compactness. Figure 3d illustrates representative prune candidates. The proposed criterion considers point support, local coverage, neighborhood density, spatial proximity, and optimization status.

Let $I_{\mathrm{low}}$ denote the index set of Gaussian primitives whose gradient magnitudes belong to the lowest $\rho_{g}$ fraction of the current Gaussian set. The low-gradient indicator is defined as

(39) $$B_{i}=\left\{\begin{array}{cc} 1, & i\in I_{\mathrm{low}},\\ 0, & \mathrm{otherwise}. \end{array}\right.$$

A Gaussian primitive $G_{i}$ is selected for pruning when

(40) $$\mathrm{Prune}\left(G_{i}\right)=\left\{\begin{array}{cc} 1, & L_{i}\vee Q_{i}\vee D_{i}\vee R_{i},\\ 0, & \mathrm{otherwise}, \end{array}\right.$$

where

(41) $$\begin{array}{cc} L_{i} & =(n_{i}^{\sup}\leq \tau_{L}),\\ Q_{i} & =\left(n_{i}^{3\sigma }<\tau_{p}\right)\wedge \left(B_{i}=1\right),\\ D_{i} & =\left(N_{i}^{2.5s_{\max}}\geq \tau_{d}\right)\wedge \left(B_{i}=1\right),\\ R_{i} & =\left(N_{i}^{0.5m}\geq \tau_{r}\right)\wedge \left(B_{i}=1\right). \end{array}$$

Here, $n_{i}^{\sup}$ is the number of input points within the support region used for point-to-Gaussian association. The quantity $n_{i}^{3\sigma }$ is the number of input points within a sphere of radius $3s_{i}^{\max}$ centered at $\mu_{i}$, where $s_{i}^{\max}=\max\left(s_{i}\right)$. The quantities $N_{i}^{2.5s_{\max}}$ and $N_{i}^{0.5m}$ denote the numbers of neighboring Gaussian primitives, excluding $G_{i}$, within radii $2.5s_{i}^{\max}$ and 0.5 m, respectively.

The condition $L_{i}$ directly removes Gaussian primitives with insufficient point support, whereas $Q_{i}$, $D_{i}$, and $R_{i}$ remove poorly covered or locally redundant primitives only when they also belong to the low-gradient set. Thus, a small gradient alone does not trigger pruning.

Pruning begins after iteration 70 and is applied every 110 iterations with a 55-iteration offset. This delayed schedule prevents premature removal while reducing redundant primitives and preserving localization-relevant geometry.

When a Gaussian primitive satisfies the pruning condition, it is removed from the Gaussian set following the pruning operation of 3DGS [11]. The remaining primitives are retained and further refined in subsequent optimization iterations and are used for localization.

#### 3.5.5. Create-in-Learning

Even when split, clone, merge, and prune operations are repeatedly applied during optimization, some regions may remain uncovered by the support regions of existing Gaussian primitives. These regions contain input points but lack sufficient Gaussian representation, potentially resulting in the loss of local geometric information required for localization. To address this issue, the proposed framework incorporates an adaptive Gaussian creation strategy that generates new Gaussian primitives during optimization.

**Figure 4:**
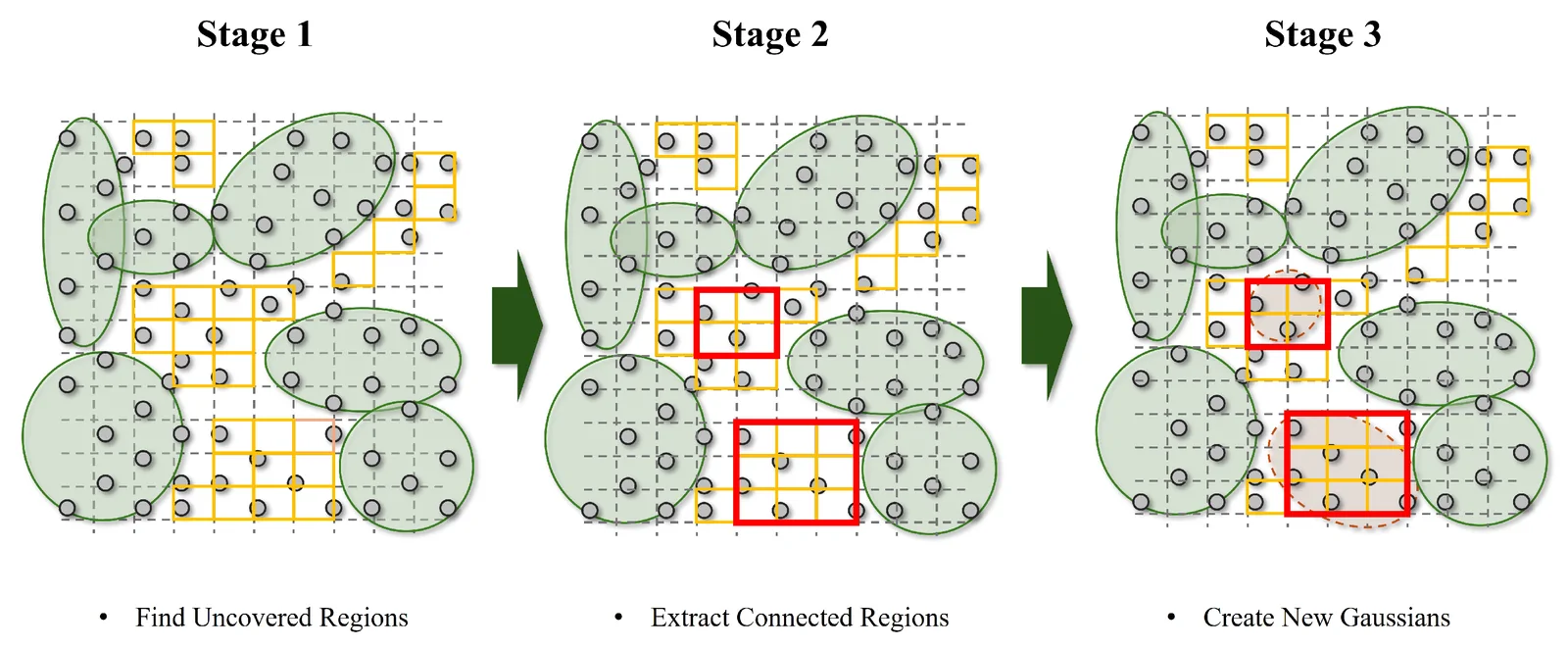
Create-in-learning operation. Uncovered point-supported regions are identified, grouped, and initialized as new Gaussian primitives.

Figure 4 illustrates the adaptive Gaussian creation procedure. First, the entire space is partitioned into a voxel grid with voxel size $\ell_{\mathrm{vox}}$, and the points contained within each voxel are identified. Voxels containing input points but not covered by the support region $\Omega_{i}$ of any existing Gaussian primitive are defined as uncovered regions:

(42) $$V^{\mathrm{unc}}=\left\{v\;|\;N\left(v\right)>0\;\wedge \;v\notin \underset{i}{⋃}\Omega_{i}\right\}$$

where N(v) denotes the number of points contained in voxel *v*, and $\Omega_{i}$ denotes the support region of the Gaussian primitive $G_{i}$ used for coverage checking. In this study, $\Omega_{i}$ is defined using a support-region scale factor $\sigma_{\mathrm{cover}}$.

Adjacent uncovered voxels are then grouped into connected components:

(43) $$V_{\mathrm{connect}}=\left\{V^{1},V^{2},\ldots ,V^{N_{\mathrm{cc}}}\right\}.$$

For each connected component, the points contained within the corresponding voxel set are collected, and a new Gaussian primitive is initialized using the mean and covariance estimated from the collected points. To avoid generating Gaussian primitives from isolated sparse points or noise-induced uncovered voxels, a new primitive is created only when the number of collected points exceeds a minimum point threshold $N_{\min}^{\mathrm{create}}$.

When a connected uncovered region satisfies this creation condition, a new Gaussian primitive is generated using the same point-statistics-based initialization procedure described in Section 3.3. The newly created primitive is inserted into the Gaussian set and refined in subsequent optimization iterations.

In the optimization process, the create-in-learning operation is scheduled from iteration 50 and is applied every 100 iterations. This periodic creation step allows point-supported but under-represented regions to be incorporated during optimization.

Overall, the adaptive operations are applied according to predefined iteration schedules rather than being executed simultaneously at every iteration. The split and clone operations are scheduled with different offsets to avoid simultaneous candidate conflicts. The merge and prune operations are delayed until the Gaussian distribution becomes sufficiently stable. Create-in-learning is periodically applied to recover uncovered point-supported regions. The entire optimization process consists of 1100 iterations. Geometry-aware adaptive Gaussian control is performed during the first 1000 iterations, and the remaining 100 iterations are used for parameter refinement using only the geometry-aware optimization losses. This final refinement stage stabilizes the Gaussian parameters after the structural updates.

## 4. Experiment

This section quantitatively evaluates the proposed geometry-aware adaptive Gaussian mapping framework. The evaluation focuses on map compression efficiency and localization performance to assess the effectiveness of the proposed Gaussian map representation.

### 4.1. Experimental Setup

#### 4.1.1. Dataset and Comparison Protocol

Experiments were conducted on the KITTI-360 dataset [35]. For map construction, we used the accumulated static 3D point clouds provided in the KITTI-360 semantic annotations. This allows all methods to be constructed from the same pre-built static map input, enabling a controlled comparison focused on the effect of the map representation itself. All map representations were constructed from the same static point-cloud inputs and evaluated on five KITTI-360 sequences: 00, 02, 03, 04, and 05. For each sequence, an identical 60-s driving segment was used for localization evaluation with the corresponding LiDAR scans.

The proposed LGM was compared with three representative LiDAR map representations: the conventional NDT map, VoxelMap [22], and Splat-LOAM [31]. NDT was selected as a standard probabilistic voxel-based baseline. VoxelMap was selected as an enhanced voxel representation with local geometric modeling, and Splat-LOAM as a recent Gaussian-based mapping method. These baselines were chosen because they produce probabilistic or Gaussian-compatible map representations from LiDAR point clouds, enabling a controlled comparison of map compactness and localization performance.

For localization, the NDT baseline was evaluated using its conventional registration pipeline. In contrast, VoxelMap, Splat-LOAM, and the proposed LGM were evaluated using the same point-to-Gaussian registration framework described in [36], after converting their map outputs into an equivalent Gaussian parameterization. NDT registration relies on a fixed-voxel structure. Therefore, replacing its native pipeline with the grid-free registration used for LGM would alter its underlying registration formulation. Consequently, the NDT–LGM comparison reflects both map-representation and registration-backend differences, which limits the isolation of the map representation alone. Nevertheless, NDT remains a representative Gaussian-map baseline for quantitatively assessing whether LGM provides reliable and competitive localization performance.

#### 4.1.2. Hardware Environment

The map-construction experiments were conducted on a server equipped with two Intel Xeon Gold 5416S CPUs, 512 GB of system memory, and four NVIDIA RTX PRO 6000 GPUs with 96 GB of memory each. The localization experiments were conducted on a separate system equipped with an Intel Xeon W-11855M CPU and 128 GB of system memory. All localization methods were evaluated on the CPU without GPU acceleration.

#### 4.1.3. Evaluation Metrics

Map compression performance was evaluated using the data size (kB), which is the actual file size of the saved map used for localization. To ensure a fair comparison, all map representations were stored using the same parameter format. Each Gaussian primitive was represented by ten float32 parameters, consisting of the mean (3), scale (3), and rotation represented by a quaternion (4). For planar Gaussian representations, the missing out-of-plane scale field was assigned a small constant value of $10^{-3}$. This field was included in our reconstructed representation to avoid underestimating the storage size of planar Gaussian maps due to missing out-of-plane scale parameters. Although the magnitude of this value had a negligible effect on the file size, a nonzero value was used to prevent singular covariance matrices when the converted representation is used for localization.

Localization performance was evaluated using translation and heading error, which are the commonly adopted metrics in LiDAR-based localization studies. Lateral and longitudinal errors were measured to provide a more detailed analysis of localization performance with respect to the direction of motion of the vehicle.

Translation error was computed as the mean absolute error (MAE) of the three-dimensional Euclidean distance between the estimated and ground-truth poses. Lateral and longitudinal errors represent the position errors perpendicular and parallel to the heading direction of the vehicle, respectively. Heading error was defined as the yaw angle difference between the estimated and ground-truth orientations.

Peak map-loading memory was measured as the maximum increase in the process resident set size (RSS) observed from immediately before loading each map until the map had been fully loaded and prepared for localization. The process memory measured before map loading was used as the baseline, allowing the additional CPU memory consumed during the map-loading stage to be quantified.

Serialized map size measures storage and transmission requirements, whereas peak map-loading memory measures the maximum additional resident memory during map loading and initialization. The reduction in serialized map size therefore does not imply a proportional reduction in memory usage, which is evaluated separately.

These metrics enable a quantitative comparison of localization accuracy, map compactness, and map-loading memory efficiency among different maps. In addition to the serialized map size, the number of Gaussian primitives and peak map-loading memory were reported to evaluate both the compactness of each map and the memory required to load it. The parameter settings used in the proposed framework are summarized in Table A1 of Appendix A.

### 4.2. Performance Analysis

#### 4.2.1. Map Compression Analysis

This section evaluates the map compression performance of the proposed Gaussian mapping framework. Table 1 compares the map storage size and the number of Gaussian primitives among the conventional NDT map, VoxelMap, Splat-LOAM, and the proposed LGM on different KITTI-360 sequences.

As shown in Table 1, the proposed LGM consistently achieves the smallest map storage size across all evaluated sequences. The average storage size is reduced from 938.9 kB for the conventional NDT map to 374.7 kB, an average reduction of approximately 60%. Although VoxelMap was not originally designed for map compression, it still provides a certain degree of storage reduction compared with NDT, achieving an average storage size of 756.9 kB. In contrast, Splat-LOAM requires substantially larger storage because it is primarily designed to construct dense Gaussian representations for accurate geometric and depth rendering rather than compact map generation.

A similar trend can be observed in the number of Gaussian primitives. The proposed LGM contains only 5449–14,065 Gaussian primitives, whereas NDT requires 22,103–24,815 primitives. VoxelMap reduces the number of primitives compared with NDT, whereas Splat-LOAM maintains significantly denser Gaussian representations to preserve fine geometric details. These results indicate that the proposed method generates a substantially more compact Gaussian map representation than the existing approaches.

The compact map size achieved by the proposed method arises not simply from the use of Gaussian primitives, but from the joint operation of geometry-aware optimization and adaptive Gaussian control. The geometry-aware loss aligns each primitive with the local structure, while the adaptive Gaussian control regulates its number and distribution according to geometric complexity. Without being restricted to a fixed voxel partition, the proposed framework reduces redundant representation while preserving localization-relevant geometry, resulting in a compact Gaussian map.

Overall, the proposed geometry-aware adaptive Gaussian mapping framework achieves the highest map compression efficiency among the evaluated methods while maintaining a map representation suitable for LiDAR scan-matching localization. These results demonstrate that explicitly incorporating geometric awareness into the Gaussian optimization process is an effective strategy for constructing compact LGMs suitable for large-scale localization.

However, a compact map representation alone does not guarantee reliable localization. Therefore, the following experiments evaluate whether the proposed LGM can preserve localization performance despite the substantial reduction in map complexity.

#### 4.2.2. Localization Performance

This section evaluates the localization performance of the proposed LGM and compares it with representative map representations, including the conventional NDT, VoxelMap, and Splat-LOAM.

##### Trajectory-Averaged Localization Performance

To quantitatively evaluate the overall localization performance, trajectory-averaged localization errors and scan-matching execution times were computed over five KITTI-360 sequences, namely Sequences 00, 02, 03, 04, and 05. CPU peak map-loading memory was additionally measured to evaluate the memory required to load each map for localization. Table 2 summarizes the multi-sequence comparison among NDT, VoxelMap, Splat-LOAM, and the proposed LGM.

As shown in Table 2, the proposed LGM achieves the best average performance across all evaluated metrics. Compared with NDT, LGM reduces the average translation error from 0.2712 m to 0.1681 m, the lateral error from 0.0689 m to 0.0478 m, the longitudinal error from 0.2130 m to 0.1108 m, and the heading error from 0.2091° to 0.1128°. These results indicate that the proposed compact Gaussian map preserves localization-relevant geometric constraints more effectively than the fixed-resolution NDT representation.

Compared with VoxelMap and Splat-LOAM, the proposed LGM also shows lower average localization errors while requiring substantially shorter scan-matching time. The average matching time of LGM is 27.66 ms, whereas VoxelMap and Splat-LOAM require 210.00 ms and 338.89 ms, respectively. This result shows that the proposed geometry-aware Gaussian allocation improves the efficiency of point-to-Gaussian correspondence search and subsequent pose optimization.

Across the five sequences, LGM required 77.41–82.86 MB and achieved the lowest average peak CPU memory during map loading, at 79.25 MB. This low memory requirement reflects its compact set of Gaussian primitives, which reduces the amount of map data loaded into CPU memory. Together with its compact serialized map size, these results demonstrate the memory efficiency of LGM during map loading.

Although some baselines perform better on individual metrics, LGM achieves the best average localization accuracy, matching time, and peak map-loading memory. These results indicate that LGM provides a compact and memory-efficient map while preserving the geometric constraints required for accurate and efficient LiDAR scan matching.

##### Time-Series Localization Analysis

**Figure 5:**
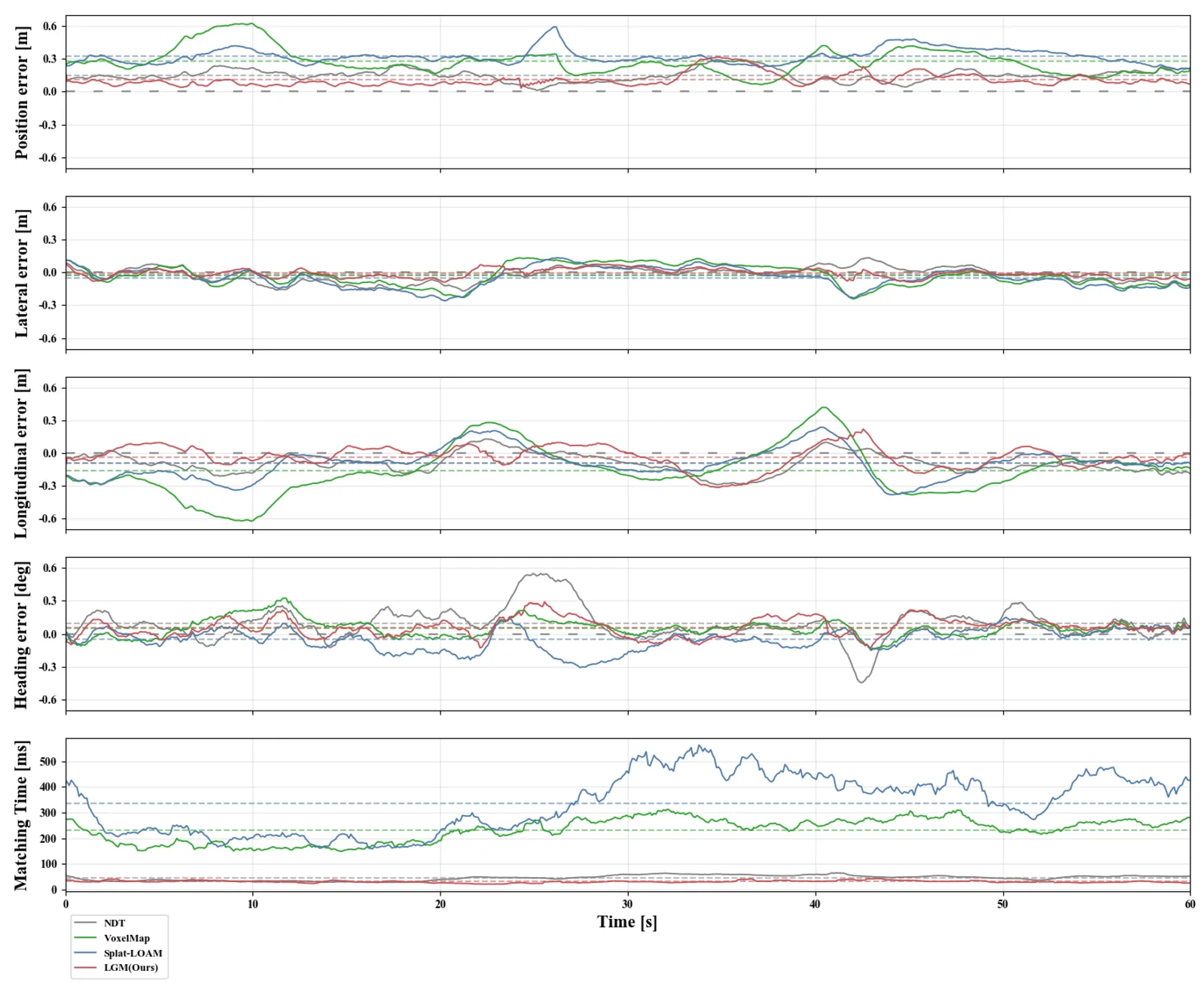
Time−series localization errors and scan-matching execution time on KITTI-360 Sequence 00. The solid curves represent the localization results of the conventional NDT, VoxelMap, Splat-LOAM, and the proposed LGM, while the dashed lines indicate the corresponding trajectory-averaged values.

Table 2 reports trajectory-averaged localization performance across multiple sequences, but averaged values do not show how localization errors and scan-matching time vary along the trajectory. To complement this averaged comparison, Figure 5 presents the frame-wise localization errors and scan-matching execution time on KITTI-360 Sequence 00. The figure illustrates the temporal variation in localization errors and scan-matching execution time for the four evaluated map representations.

Overall, the proposed LGM exhibits more stable localization behavior than the conventional NDT, VoxelMap, and Splat-LOAM. In particular, it shows smaller fluctuations in translation and lateral errors, while maintaining competitive longitudinal and heading accuracy. The translation error remains within approximately 0.0326–0.3170 m throughout the trajectory, indicating stable localization under varying driving conditions.

The advantage of the proposed framework is also observed in scan-matching time. Although NDT provides fast registration through its voxel-based representation, VoxelMap and Splat-LOAM require longer computation times because the point-to-Gaussian localization framework must search a larger set of Gaussian primitives. In contrast, the proposed LGM maintains a bounded execution time of approximately 19.14–75.01 ms over the trajectory. This is attributed to the compact and geometry-aware Gaussian representation, which reduces the point-to-Gaussian correspondence search while preserving localization-relevant geometric constraints.

Overall, the time-series results show that the proposed LGM maintains stable frame-wise localization behavior on the representative trajectory. This analysis complements the trajectory-averaged results by showing that the localization performance of LGM is maintained along the sequence, rather than being reflected only in averaged metrics.

#### 4.2.3. Qualitative Analysis of Local Gaussian Representation

**Figure 6:**
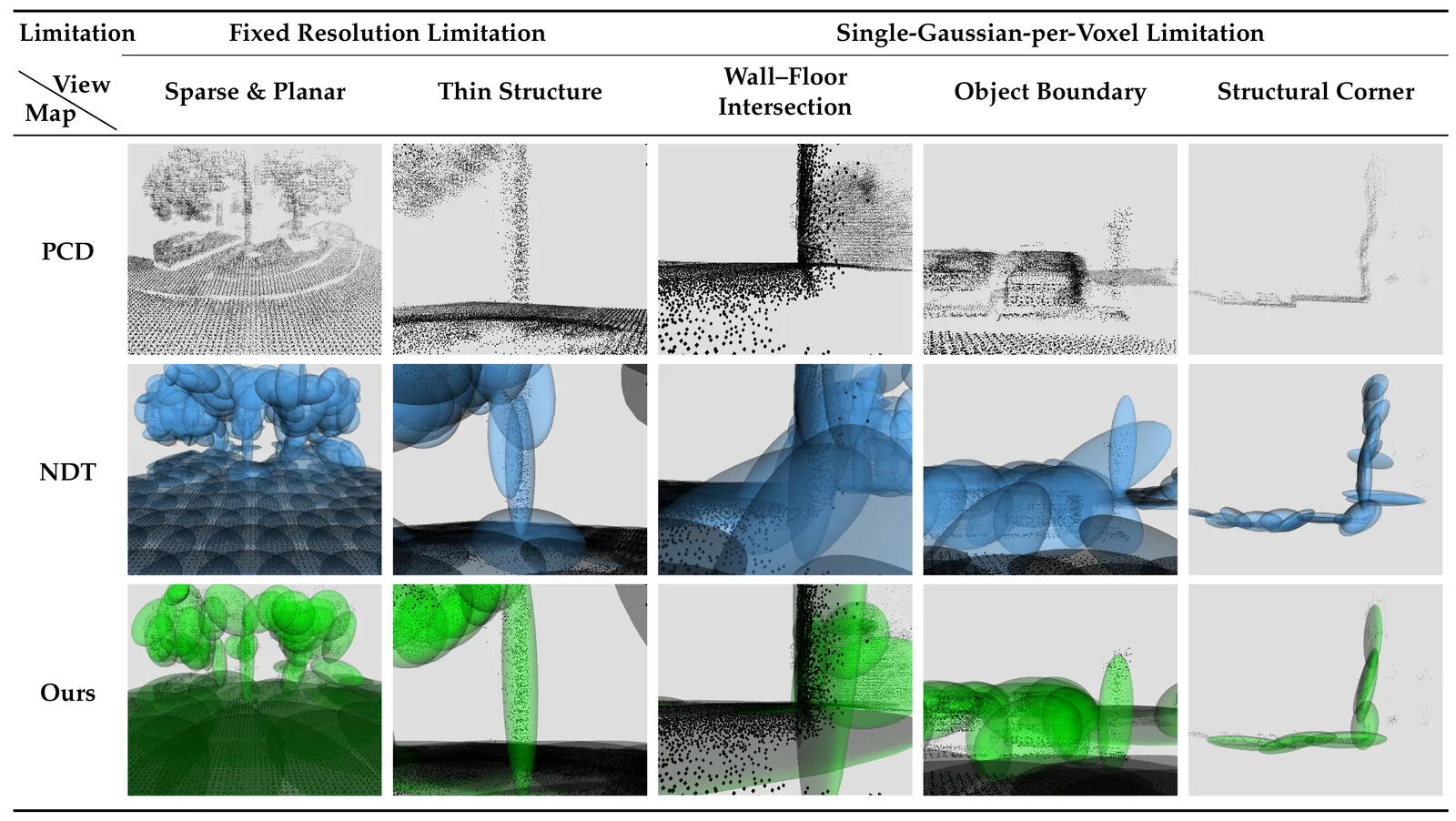
Qualitative comparison of local Gaussian representations among the PCD map, NDT map, and the proposed LGM. The examples are grouped by two representative NDT limitations: fixed voxel discretization, including fixed-resolution allocation and hard voxel-boundary effects, and single-Gaussian-per-voxel representation. The proposed LGM shows more compact primitive allocation while preserving localization-relevant local structures.

The trajectory-level and time-series localization results characterize the downstream scan-matching performance of each map. To complement these results from a local representation perspective, we examine how Gaussian primitives are distributed around representative structures such as sparse regions, boundaries, and corners. Figure 6 compares the PCD map, NDT map, and the proposed LGM. The examples are grouped according to two representative limitations of NDT: fixed voxel discretization and single-Gaussian-per-voxel representation.

The first group illustrates the limitations of fixed voxel discretization in sparse/planar and thin-structure regions. NDT’s fixed global resolution produces voxel-wise Gaussians across sparse tree regions, floor surfaces, and thin stems, potentially allocating redundant primitives where fewer would provide sufficient localization-relevant constraints. Independently estimated statistics within each voxel can also introduce boundary artifacts, representing continuous structures crossing voxel boundaries with discontinuous or redundant Gaussians. In contrast, LGM is not constrained by voxel boundaries and adaptively refines Gaussian position, scale, and orientation according to local geometric complexity, while AGC reduces unnecessary primitive allocation.

The second group illustrates the single-Gaussian-per-voxel limitation using wall–floor intersections, object boundaries, and structural corners. In these regions, multiple local surface directions or neighboring structures can coexist within the same voxel. As a result, a single NDT Gaussian may span different surfaces or objects, such as the floor and wall, a parked vehicle and adjacent structure, or a curved structural corner. The proposed LGM alleviates this limitation by refining the position, scale, and orientation of Gaussian primitives through geometry-aware losses and by adjusting primitive allocation through AGC.

Overall, the qualitative results show that the proposed framework constructs a compact Gaussian map by reducing redundant primitives in simple or sparse regions while retaining localization-relevant local structures around intersections, boundaries, and corners. These results support the main objective of the proposed LGM: reducing the number of Gaussian primitives and map size while preserving localization-relevant geometric constraints required for LiDAR localization.

#### 4.2.4. Trade-Off Analysis

##### Memory–Localization Accuracy Trade-Off

To justify the map resolution used in the main experiments and to analyze the relationship between map compactness and localization performance, a memory–localization accuracy trade-off experiment was performed. NDT maps with different voxel sizes were constructed, whereas the proposed LGM was evaluated under three Gaussian density settings: Compact, Default, and Dense. Default corresponds to the configuration used in the main experiments. Compact and Dense were used only for this trade-off analysis, with modified AGC execution intervals, offsets, and total iteration counts, as detailed in Table A2 of Appendix A. All configurations were evaluated using the same localization protocol.

Table 3 shows the trade-off between map storage, localization accuracy, and scan-matching time. For NDT, decreasing the voxel size generally improves localization accuracy by retaining finer local details, but it significantly increases the map size. In particular, the 0.5 m NDT map achieves a translation error of 0.1177 m, but requires 5615.8 kB of storage. In contrast, the 1.5 m NDT setting, which was used as the default setting in the main experiments, provides the fastest matching time of the tested NDT configurations, but its localization accuracy is lower than that of the denser NDT map.

For the proposed LGM, the trade-off between map size, localization accuracy, and scan-matching time is also observed. The compact setting achieves the smallest map size of 257.2 kB, whereas the dense setting provides the best localization accuracy with a translation error of 0.0859 m. The default setting provides a balanced configuration, achieving a map size of 562.9 kB, a translation error of 0.1091 m, and a matching time of 30.71 ms.

The advantage of the proposed LGM is particularly clear under comparable map-size conditions. The NDT map with a 2.0 m voxel size and the Default LGM require almost the same storage, 562.3 kB and 562.9 kB, respectively, and have nearly the same number of primitives. However, the proposed LGM reduces the translation error from 0.2374 m to 0.1091 m and the matching time from 43.25 ms to 30.71 ms. Conversely, when NDT uses a dense 0.5 m voxel size to achieve localization accuracy comparable to that of default LGM, its map size increases to 5615.8 kB, which is approximately ten times larger than that of the default LGM.

These results indicate that the proposed geometry-aware Gaussian representation preserves sufficient localization-relevant geometric information with fewer primitives than the conventional voxel-based NDT representation. Therefore, the proposed LGM achieves a favorable balance between map compactness and localization accuracy, rather than relying on a single favorable parameter setting.

##### Offline Map Construction Cost

In addition to online scan-matching latency, we report the offline map construction cost to clarify the practical trade-off of the proposed framework. The proposed LGM is designed for offline prior-map construction rather than real-time online mapping. Therefore, the geometry-aware optimization and adaptive Gaussian control pipeline is executed once during the map generation stage. After construction, the generated compact LGM is reused for subsequent LiDAR scan-matching localization without additional map optimization.

Table 4 reports the one-time offline map construction time and peak memory usage on KITTI-360 Sequence 00. All measurements were obtained on the same server using the native execution backend of each method. NDT and VoxelMap were executed on the CPU, whereas Splat-LOAM and the proposed LGM used GPU acceleration. Accordingly, the reported peak memory represents CPU RAM for the CPU-based methods and GPU VRAM for the GPU-based methods. These measurements characterize the implementation-level construction cost of each method on its native backend.

As shown in Table 4, the proposed LGM requires a substantially longer construction time and uses 2.251 GB of peak GPU memory because it performs iterative geometry-aware optimization and adaptive Gaussian control. This cost is incurred once during offline prior-map generation, after which the constructed compact Gaussian map can be repeatedly used for online scan-matching localization.

This makes the framework suitable for applications where prior maps are constructed offline and reused over many localization runs.

### 4.3. Ablation Study

To evaluate the contribution of each component in the proposed geometry-aware adaptive Gaussian mapping framework, ablation studies were conducted in two aspects. First, to evaluate the contribution of each loss component, we removed different combinations of the three core loss terms, i.e., geometry loss (Geo), structure loss (Struct), and surface loss (Surf), while keeping AGC unchanged. Second, to evaluate the contribution of AGC beyond the loss design, an additional ablation study was conducted by comparing the full model with a configuration in which AGC was disabled while retaining the same geometry-aware loss terms.

#### 4.3.1. Loss Ablation

As shown in Table 5, when Geo loss was removed, as in Configurations (a), (b), and (c), only a small number of Gaussian primitives survived the optimization process, resulting in extremely sparse map representations that failed to preserve sufficient geometric information for localization. Consequently, localization could not be performed without Geo. Gaussian primitives cannot establish reliable geometric correspondence with the input point cloud, causing many primitives to receive insufficient support during optimization and to be eventually removed by the pruning process. These results demonstrate that Geo loss provides the fundamental geometric constraint for Gaussian map construction and is indispensable in generating localizable map representations.

Configuration (d), which retained only Geo, remained localizable but exhibited substantially degraded localization accuracy and increased scan-matching time compared with the full model. Although geometric alignment alone can generate a valid Gaussian map, the resulting representation lacks sufficient structural regularity and local geometric consistency, leading to less efficient correspondence estimation and reduced localization robustness. This result indicates that Geo alone is insufficient for constructing an effective Gaussian representation for LiDAR localization.

Removing Struct, as in Configuration (e), further demonstrates the importance of structural regularization. Although the resulting map contains fewer Gaussian primitives and requires less storage than the full model, both localization accuracy and scan-matching efficiency deteriorate. This observation indicates that localization performance is not determined solely by the number of Gaussian primitives. Instead, Struct promotes a spatially balanced Gaussian distribution that improves point-to-Gaussian correspondence estimation and enables more efficient nonlinear pose optimization.

Configuration (f), which excludes Surf while retaining Geo and Struct, achieves localization performance close to that of the full model, indicating that Geo and Struct constitute the primary components for constructing an effective Gaussian map. Nevertheless, incorporating Surf consistently improves all localization metrics while simultaneously reducing the map size and scan-matching time. These results indicate that Surf refines the surface-related parameters of Gaussian primitives, allowing the optimization process to produce a more compact representation without sacrificing the geometric constraints required for localization.

Overall, the ablation study demonstrates that the three loss components play complementary roles. Geo establishes reliable geometric alignment with the input point cloud, Struct organizes Gaussian primitives into an efficient and localization-friendly representation, and Surf further refines local surface geometry to improve map compactness, localization accuracy, and scan-matching efficiency. Only the joint optimization of all three loss components achieves the best overall balance between map compression and LiDAR localization performance.

#### 4.3.2. Adaptive Gaussian Control Ablation

To evaluate the contribution of AGC, we compared the full model with a w/o AGC configuration. In the w/o AGC configuration, the same geometry-aware loss terms were retained, but all adaptive Gaussian control operations, including split, clone, merge, prune, and create-in-learning, were disabled. Therefore, the number of Gaussian primitives remained fixed after initialization. This setting evaluates whether geometry-aware optimization alone is sufficient, or whether adaptive primitive control is necessary for compact and localizable Gaussian map construction.

As shown in Table 6, disabling AGC produces a much smaller map, reducing the map size from 562.9 kB to 66.6 kB and the Gaussian count from 14,065 to 1657. However, this smaller map also exhibits substantially degraded localization performance, showing that a lower Gaussian count alone does not ensure an effective localization map. The translation error increases from 0.1091 m to 0.4715 m, and the lateral and longitudinal errors increase from 0.0340 m and 0.0855 m to 0.1551 m and 0.2807 m, respectively. The heading error also increases from 0.0833° to 0.2068°, and the scan-matching time increases from 30.71 ms to 39.15 ms.

These results suggest that optimizing the fixed initial Gaussian allocation alone is insufficient under the evaluated setting. The full model can increase local representational capacity through split, clone, and create-in-learning operations, while merge and prune operations remove redundant or weakly supported primitives. AGC therefore dynamically controls Gaussian count and spatial allocation to balance map compactness with the geometric constraints required for localization.

However, the full model contains 14,065 Gaussians compared with 1657 in the w/o AGC configuration, so its improved localization performance may partly reflect the increased representational capacity. This ablation therefore evaluates the overall benefit of AGC, including its adjustment of Gaussian count, without isolating the benefit of adaptive allocation at a matched primitive count.

## 5. Conclusions

This paper proposed a geometry-aware adaptive Gaussian mapping framework for LiDAR-based localization. The proposed method converts input point clouds into a set of Gaussian primitives and iteratively refines their positions, scales, orientations, and spatial distribution through geometry-aware optimization and adaptive Gaussian control. By avoiding fixed spatial discretization, the framework aims to preserve localization-relevant geometric structures while maintaining a compact map representation.

The proposed geometry-aware optimization jointly considers point-to-Gaussian alignment, structural compactness, and local surface characteristics during Gaussian map construction. In addition, adaptive Gaussian control dynamically adjusts the number and distribution of Gaussian primitives through split, clone, merge, prune, and create-in-learning operations. These operations increase representational capacity in geometrically complex or insufficiently covered regions while reducing weakly supported and redundant primitives. Consequently, the proposed framework allocates Gaussian primitives according to local geometric complexity rather than uniformly increasing map resolution.

Experiments on the KITTI-360 dataset showed that the proposed LGM reduced map storage requirements by approximately 60% on average, with a maximum reduction of 75.7%, while maintaining localization accuracy comparable to or better than that of the conventional NDT localization pipeline. Under a common point-to-Gaussian localization framework, the proposed LGM achieved the lowest translation, lateral, and longitudinal errors of the evaluated Gaussian-based maps, together with competitive heading accuracy and substantially lower scan-matching time. The memory–localization accuracy trade-off analysis further showed that the proposed representation maintains favorable localization performance under comparable map-size conditions, indicating that geometry-aware Gaussian allocation uses the available map capacity more effectively than fixed-resolution voxel-based representations. The ablation study also confirmed that the geometric, structural, and surface constraints jointly contribute to compact and localizable Gaussian map generation.

Overall, the proposed framework demonstrates that geometry-aware Gaussian optimization can provide a favorable balance among map compactness, localization accuracy, online computational efficiency, and map-loading memory efficiency while preserving the geometric constraints required for LiDAR localization under the evaluated static-map setting. The main contribution of this study is therefore not limited to map-size reduction, but also includes an adaptive Gaussian map construction strategy that preserves localization-relevant geometry with a substantially smaller number of primitives.

The current framework assumes a static environment during map construction and localization, and therefore does not explicitly address dynamic objects or long-term environmental changes. Future work will investigate dynamic Gaussian map representations, adaptive map updating, and lifelong mapping strategies to maintain accurate and compact maps in continuously evolving environments.

## Figures and Tables

**Table 1:** Comparison of map storage size and the number of Gaussian primitives among NDT, VoxelMap, Splat-LOAM and the proposed LGM on KITTI-360 sequences. In this and subsequent tables, bold values indicate the best performance in each metric.

Metric	Type	00	02	03	04	05
Data Size (kB)	NDT	960.6	958.6	992.9	884.4	897.8
	VoxelMap	667.7	635.2	951.8	817.8	712.0
	Splat-LOAM	14,175.4	17,705.1	30,644.9	18,524.0	18,893.3
	LGM (Ours)	**562.9**	**514.6**	**241.2**	**336.5**	**218.2**
Primitives	NDT	24,007	23,957	24,815	22,103	22,437
	VoxelMap	16,686	15,874	23,788	20,439	17,792
	Splat-LOAM	354,379	442,621	766,118	463,095	472,327
	LGM (Ours)	**14,065**	**12,857**	**6023**	**8399**	**5449**

**Table 2:** Multi-sequence comparison of localization accuracy, scan-matching time, and peak CPU map-loading memory usage among NDT, VoxelMap, Splat-LOAM, and the proposed LiDAR Gaussian Map (LGM) on KITTI-360 Sequences 00, 02, 03, 04, and 05.

Seq.	Map	Translation (m) ↓	Lateral (m) ↓	Longitudinal (m) ↓	Heading (°) ↓	Matching (ms) ↓	Memory (MB) ↓
00	NDT	0.1534	0.0617	0.1063	0.1369	**25.54**	86.63
	VoxelMap	0.2743	0.0851	0.2277	**0.0746**	259.64	83.96
	Splat-LOAM	0.3053	0.0839	0.1286	0.0990	348.52	207.34
	LGM (Ours)	**0.1091**	**0.0340**	**0.0855**	0.0833	30.71	**82.86**
02	NDT	0.2540	0.0684	0.1517	0.2283	**27.94**	82.81
	VoxelMap	0.3428	0.0854	0.1152	0.1603	141.33	81.36
	Splat-LOAM	0.1513	0.0433	**0.0658**	0.1102	421.81	237.23
	LGM (Ours)	**0.1122**	**0.0320**	0.0661	**0.0833**	29.87	**80.19**
03	NDT	0.2379	0.0538	0.2208	0.3765	24.32	90.31
	VoxelMap	0.2791	0.0682	0.2313	0.1948	226.48	86.07
	Splat-LOAM	0.2333	**0.0519**	**0.1824**	0.2840	338.97	356.27
	LGM (Ours)	**0.2317**	0.0711	0.2002	**0.1076**	**22.87**	**77.41**
04	NDT	0.4387	0.0716	0.3872	0.1486	37.06	83.61
	VoxelMap	0.3712	0.1007	0.1453	0.1641	241.14	84.08
	Splat-LOAM	0.2691	0.1416	0.1606	**0.1394**	294.17	245.03
	LGM (Ours)	**0.2046**	**0.0537**	**0.0901**	0.1498	**26.13**	**78.27**
05	NDT	0.2719	0.0889	0.1991	0.1551	41.46	83.65
	VoxelMap	**0.1647**	0.0723	0.1317	**0.0618**	181.42	81.90
	Splat-LOAM	0.2436	0.0528	0.1423	0.0849	290.96	248.56
	LGM (Ours)	0.1829	**0.0484**	**0.1123**	0.1400	**28.71**	**77.54**
Avg	NDT	0.2712	0.0689	0.2130	0.2091	31.26	85.40
	VoxelMap	0.2864	0.0823	0.1702	0.1311	210.00	83.47
	Splat-LOAM	0.2405	0.0747	0.1359	0.1435	338.89	258.89
	LGM (Ours)	**0.1681**	**0.0478**	**0.1108**	**0.1128**	**27.66**	**79.25**

**Table 3:** Memory–localization accuracy trade-off analysis of NDT map and the proposed LGM on KITTI-360 Sequence 00 under different voxel sizes for NDT and Gaussian density settings.

Map	Configuration	Map Compression		Localization				
		Map Size (kB) ↓	Primitive Count ↓	Translation (m) ↓	Lateral (m) ↓	Longitudinal (m) ↓	Heading (°) ↓	Matching (ms) ↓
NDT	0.5 m	5615.8	140,389	0.1177	0.0366	0.0957	0.0947	37.72
	1.0 m	1942.0	48,545	0.1439	0.0382	0.1241	0.1173	40.29
	1.5 m ^†^	960.6	24,007	0.1534	0.0617	0.1063	0.1369	**25.54**
	2.0 m	562.3	14,052	0.2374	0.1003	0.1639	0.1533	43.25
	2.5 m	417.6	10,435	0.5786	0.1668	0.3097	0.2159	40.50
Ours	Compact	**257.2**	**6418**	0.1137	0.0456	0.0827	0.0978	28.61
	Default ^†^	562.9	14,065	0.1091	0.0340	0.0855	0.0833	30.71
	Dense	931.0	23,267	**0.0859**	**0.0334**	**0.0680**	**0.0695**	44.13

Note: Bold values indicate the best performance in each metric. The superscript ^†^ indicates the default setting used in the main experiments.

**Table 4:** One-time offline map construction cost of NDT, VoxelMap, Splat-LOAM, and the proposed LGM on KITTI-360 Sequence 00. Construction time was measured on the native execution backend of each method.

Map	Backend	Construction Time	Peak Memory (GB)
NDT	CPU	1.2 s	0.972
VoxelMap	CPU	1 min 0.5 s	0.225
Splat-LOAM	GPU	21 min 49.6 s	1.533
Ours	GPU	5 h 36 min 4.1 s	2.251

**Table 5:** Ablation study results for different loss configurations evaluated on KITTI-360 Sequence 00. Loss components marked with ✓ are included in the corresponding configuration.

Config	Loss Components			Map Compression		Localization				
	$L_{\mathrm{Geo}}$	$L_{\mathrm{Struct}}$	$L_{\mathrm{Surf}}$	Map Size (kB) ↓	Gaussian Count ↓	Translation (m) ↓	Lateral (m) ↓	Longitudinal (m) ↓	Heading (°) ↓	Matching (ms) ↓
(a)			✓	**0.91**	**16**	*Localization Failure*				
(b)		✓		1.67	33	*Localization Failure*				
(c)		✓	✓	2.26	49	*Localization Failure*				
(d)	✓			155.5	3881	0.3757	0.2106	0.2007	0.1694	79.30
(e)	✓		✓	255.0	6368	0.2245	0.1220	0.1366	0.1463	77.31
(f)	✓	✓		596.8	14,908	0.1103	0.0406	0.0997	0.0874	36.92
Ours	✓	✓	✓	562.9	14,065	**0.1091**	**0.0340**	**0.0855**	**0.0833**	**30.71**

**Table 6:** Ablation study on Adaptive Gaussian Control evaluated on KITTI-360 Sequence 00.

Config	Map Compression		Localization				
	Map Size (kB) ↓	Gaussian Count ↓	Translation (m) ↓	Lateral (m) ↓	Longitudinal (m) ↓	Heading (°) ↓	Matching (ms) ↓
w/o AGC	**66.6**	**1657**	0.4715	0.1551	0.2807	0.2068	39.15
Ours	562.9	14,065	**0.1091**	**0.0340**	**0.0855**	**0.0833**	**30.71**

## Data Availability

The KITTI-360 dataset analyzed in this study is publicly available at https://www.cvlibs.net/datasets/kitti-360/ (accessed on 22 September 2026). The data generated during this study, including the Gaussian map data and experimental configurations, are available from the corresponding author upon reasonable request.

## Appendix A. Experimental Parameters

Unless otherwise specified, the parameter values listed in Table A1 correspond to the Default configuration and were empirically selected to balance map compactness and localization performance. These values were fixed across the main experiments.

**Table A1:** Parameter settings used in the proposed framework.

Parameter	Value	Parameter	Value
$r_{\mathrm{init}}$	1.5	$N_{\min}^{\mathrm{init}}$	3
$N_{\max}^{\mathrm{init}}$	5	$r_{\mathrm{excl}}$	3.0
$\alpha_{\mathrm{mah}}$	2.4	$\alpha_{\mathrm{ch}}$	0.5
$\beta_{\mathrm{ov}}$	0.01	$\beta_{\mathrm{like}}$	0.35
$\beta_{\mathrm{nll}}$	1.0	$\beta_{\mathrm{comp}}$	4.0
$\tau_{\mathrm{ov}}$	3.0	$\tau_{\mathrm{var}}$	$10^{-4}$
$\gamma_{\mathrm{area}}$	0.2	$n_{\sigma }$	3.0
$\tau_{g}$	0.01	$\tau_{f1}$	0.9
$\tau_{c1}$	0.9	$\tau_{f2}$	0.85
$\tau_{c2}$	0.85	$\tau_{f3}$	0.7
$\tau_{c3}$	0.7	$\tau_{s}$	2.0
$\sigma_{m}$	3.0	$K_{\mathrm{merge}}$	4
$\tau_{m}$	0.7	$\tau_{\mathrm{dir}}$	0.6
$\tau_{\mathrm{plane}}$	0.5	$\rho_{g}$	0.5
$\tau_{L}$	3	$\tau_{p}$	3
$\tau_{d}$	2	$\tau_{r}$	2
$\ell_{\mathrm{vox}}$	0.5	$\sigma_{\mathrm{cover}}$	2.0
$N_{\min}^{\mathrm{create}}$	3	ϵ	$10^{-5}$

**Table A2:** AGC scheduling parameters and optimization budgets for the Compact, Default, and Dense configurations. All values are expressed in iterations. The final 100 iterations of each configuration are reserved for parameter refinement without AGC.

Operation	Configuration	Start	Interval	Offset	Total Iterations	AGC Cutoff
Split	Compact	30	50	0	1000	900
	Default	30	40	0	1100	1000
	Dense	30	30	0	1200	1100
Clone	Compact	30	50	25	1000	900
	Default	30	40	20	1100	1000
	Dense	30	30	15	1200	1100
Merge	Compact	70	40	22	1000	900
	Default	70	60	32	1100	1000
	Dense	70	80	42	1200	1100
Prune	Compact	70	90	45	1000	900
	Default	70	110	55	1100	1000
	Dense	70	130	65	1200	1100
Create-in-learning	Compact	50	120	0	1000	900
	Default	50	100	0	1100	1000
	Dense	50	80	0	1200	1100

## References

[1] Asrat K.T., Cho H.-J. (2024). A Comprehensive Survey on High-Definition Map Generation and Maintenance. ISPRS Int. J. Geo-Inf..

[2] Charroud A., El Moutaouakil K., Palade V., Yahyaouy A., Onyekpe U., Eyo E.U. (2024). Localization and Mapping for Self-Driving Vehicles: A Survey. Machines.

[3] Gotlib D., Gartner G., Miksa K. (2026). HD Maps for Autonomous Vehicles: Implications for Cartographic Theory and Practice. ISPRS Int. J. Geo-Inf..

[4] Wu B., Xu C., Kong H. (2023). LiDAR Road-Atlas: An Efficient Map Representation for General 3D Urban Environment. Field Robot..

[5] Chen S., Liu B., Feng C., Vallespi-Gonzalez C., Wellington C. (2021). 3D Point Cloud Processing and Learning for Autonomous Driving: Impacting Map Creation, Localization, and Perception. IEEE Signal Process. Mag..

[6] Xu S., Zhao K., Sun Y., Fu X., Luo K. (2025). Visual Point Cloud Map Construction and Matching Localization for Autonomous Vehicle. Drones.

[7] Biber P., Strasser W. The Normal Distributions Transform: A New Approach to Laser Scan Matching. Proceedings of the 2003 IEEE/RSJ International Conference on Intelligent Robots and Systems.

[8] Kim C., Cho S., Sunwoo M., Resende P., Bradai B., Jo K. (2021). A Geodetic Normal Distribution Map for Long-Term LiDAR Localization on Earth. IEEE Access.

[9] Stoyanov T., Magnusson M., Andreasson H., Lilienthal A.J. (2012). Fast and Accurate Scan Registration through Minimization of the Distance between Compact 3D NDT Representations. Int. J. Robot. Res..

[10] Saarinen J.P., Andreasson H., Stoyanov T., Lilienthal A.J. (2013). 3D Normal Distributions Transform Occupancy Maps: An Efficient Representation for Mapping in Dynamic Environments. Int. J. Robot. Res..

[11] Kerbl B., Kopanas G., Leimkuehler T., Drettakis G. (2023). 3D Gaussian Splatting for Real-Time Radiance Field Rendering. ACM Trans. Graph..

[12] Yu Z., Chen A., Huang B., Sattler T., Geiger A. Mip-Splatting: Alias-free 3D Gaussian Splatting. Proceedings of the IEEE/CVF Conference on Computer Vision and Pattern Recognition.

[13] Ren K., Jiang L., Lu T., Yu M., Xu L., Ni Z., Dai B. (2024). Octree-GS: Towards Consistent Real-time Rendering with LOD-Structured 3D Gaussians. arXiv.

[14] Besl P.J., McKay N.D. (1992). A Method for Registration of 3-D Shapes. IEEE Trans. Pattern Anal. Mach. Intell..

[15] Segal A., Haehnel D., Thrun S. Generalized-ICP. Proceedings of the Robotics: Science and Systems.

[16] Wen J., Zhang X., Su Y., Li J., Zhang J. (2023). Real-Time Scan-to-Map Matching Localization System Based on Lightweight Pre-Built Occupancy High-Definition Map. Remote Sens..

[17] Gong Y., Zhang X., Feng J., He X., Zhang D. (2024). LiDAR-based HD Map Localization Using Semantic Generalized ICP with Road Marking Detection. arXiv.

[18] Yin H., Wang Y., Tang L., Ding X., Huang S., Xiong R. (2021). 3D LiDAR Map Compression for Efficient Localization on Resource Constrained Vehicles. IEEE Trans. Intell. Transp. Syst..

[19] Magnusson M. (2009). The Three-Dimensional Normal-Distributions Transform: An Efficient Representation for Registration, Surface Analysis, and Loop Detection. Ph.D. Thesis.

[20] Jang K.W., Jeong W.J., Kang Y. (2022). Development of a GPU-Accelerated NDT Localization Algorithm for GNSS-Denied Urban Areas. Sensors.

[21] Kim M., Kim J., Kim D., Kim H. (2023). Vehicle-to-Infrastructure-Based LiDAR Localization Method Using NDT Map for Autonomous Driving. Electronics.

[22] Yuan C., Xu W., Liu X., Hong X., Zhang F. (2022). Efficient and Probabilistic Adaptive Voxel Mapping for Accurate Online LiDAR Odometry. IEEE Robot. Autom. Lett..

[23] Eckart B., Kim K., Kautz J. HGMR: Hierarchical Gaussian Mixtures for Adaptive 3D Registration. Proceedings of the European Conference on Computer Vision.

[24] Li P.Z.X., Karaman S., Sze V. (2024). GMMap: Memory-Efficient Continuous Occupancy Map Using Gaussian Mixture Model. IEEE Trans. Robot..

[25] Kim T., Jeon H., Lee D. (2023). A Multi-Layered 3D NDT Scan-Matching Method for Robust Localization in Logistics Warehouse Environments. Sensors.

[26] Hornung A., Wurm K.M., Bennewitz M., Stachniss C., Burgard W. (2013). OctoMap: An Efficient Probabilistic 3D Mapping Framework Based on Octrees. Auton. Robots.

[27] Matsuki H., Murai R., Kelly P.H.J., Davison A.J. Gaussian Splatting SLAM. Proceedings of the IEEE/CVF Conference on Computer Vision and Pattern Recognition.

[28] Yan C., Qu D., Xu D., Zhao B., Wang Z., Wang D., Li X. GS-SLAM: Dense Visual SLAM with 3D Gaussian Splatting. Proceedings of the IEEE/CVF Conference on Computer Vision and Pattern Recognition.

[29] Jin L., Zhong X., Pan Y., Behley J., Stachniss C., Popović M. (2025). ActiveGS: Active Scene Reconstruction Using Gaussian Splatting. IEEE Robot. Autom. Lett..

[30] Zhai H., Zhang X., Zhao B., Li H., He Y., Cui Z., Bao H., Zhang G. (2025). SplatLoc: 3D Gaussian Splatting-Based Visual Localization for Augmented Reality. IEEE Trans. Vis. Comput. Graph..

[31] Giacomini E., Di Giammarino L., De Rebotti L., Grisetti G., Oswald M.R. Splat-LOAM: Gaussian Splatting LiDAR Odometry and Mapping. Proceedings of the IEEE/CVF International Conference on Computer Vision.

[32] Hong S., Zheng C., Shen Y., Li C., Zhang F., Qin T., Shen S. (2025). GS-LIVO: Real-Time LiDAR, Inertial, and Visual Multisensor Fused Odometry with Gaussian Mapping. IEEE Trans. Robot..

[33] Fan H., Su H., Guibas L.J. A Point Set Generation Network for 3D Object Reconstruction from a Single Image. Proceedings of the IEEE Conference on Computer Vision and Pattern Recognition.

[34] Bishop C.M. (2006). Pattern Recognition and Machine Learning.

[35] Liao Y., Xie J., Geiger A. (2023). KITTI-360: A Novel Dataset and Benchmarks for Urban Scene Understanding in 2D and 3D. IEEE Trans. Pattern Anal. Mach. Intell..

[36] Lee H.-S., Kim S.-J., Kim C.-S. (2025). Real-Time LiDAR Localization on 3D Gaussian Splatting Maps Using Hash-Map Search. J. Digit. Contents Soc..

